# Morphology, Molecules, and Monogenean Parasites: An Example of an Integrative Approach to Cichlid Biodiversity

**DOI:** 10.1371/journal.pone.0124474

**Published:** 2015-04-29

**Authors:** Maarten Van Steenberge, Antoine Pariselle, Tine Huyse, Filip A. M. Volckaert, Jos Snoeks, Maarten P. M. Vanhove

**Affiliations:** 1 Biology Department, Royal Museum for Central Africa, Tervuren, Belgium; 2 Laboratory of Biodiversity and Evolutionary Genomics, Department of Biology, University of Leuven, Leuven, Belgium; 3 Institute of Zoology, University of Graz, Graz, Austria; 4 Institut des Sciences de l'Évolution, IRD-CNRS-Université Montpellier, Montpellier, France; 5 Institute of Marine Biological Resources and Inland Waters, Hellenic Centre for Marine Research, Anavyssos, Greece; University of Colorado, UNITED STATES

## Abstract

The unparalleled biodiversity of Lake Tanganyika (Africa) has fascinated biologists for over a century; its unique cichlid communities are a preferred model for evolutionary research. Although species delineation is, in most cases, relatively straightforward, higher-order classifications were shown not to agree with monophyletic groups. Here, traditional morphological methods meet their limitations. A typical example are the tropheine cichlids currently belonging to *Simochromis* and *Pseudosimochromis*. The affiliations of these widespread and abundant cichlids are poorly understood. Molecular work suggested that genus and species boundaries should be revised. Moreover, previous morphological results indicated that intraspecific variation should be considered to delineate species in Lake Tanganyika cichlids. We review the genera *Simochromis* and *Pseudosimochromis* using an integrative approach. Besides a morphometric study and a barcoding approach, monogenean *Cichlidogyrus* (Platyhelminthes: Ancyrocephalidae) gill parasites, often highly species-specific, are used as complementary markers. Six new species are described. *Cichlidogyrus raeymaekersi* **sp. nov.**, *C*. *muterezii* **sp. nov.** and *C*. *banyankimbonai* **sp. nov.** infect *S*. *diagramma*. *Cichlidogyrus georgesmertensi* **sp. nov.** was found on *S*. *babaulti* and *S*. *pleurospilus*, *C*. *franswittei* **sp. nov.** on both *S*. *marginatus* and *P*. *curvifrons* and *C*. *frankwillemsi* **sp. nov.** only on *P*. *curvifrons*. As relatedness between *Cichlidogyrus* species usually reflects relatedness between hosts, we considered *Simochromis* monotypic because the three *Cichlidogyrus* species found on *S*. *diagramma* belonged to a different morphotype than those found on the other *Simochromis*. The transfer of *S*. *babaulti*, *S*. *marginatus*, *S*. *pleurospilus* and *S*. *margaretae* to *Pseudosimochromis* was justified by the similarity of their *Cichlidogyrus* fauna and the intermediate morphology of *S*. *margaretae*. Finally parasite data also supported the synonymy between *S*. *pleurospilus* and *S*. *babaulti*, a species that contains a large amount of geographical morphological variation.

## Introduction

Systematics is the science of the world’s biodiversity and its interrelationships. Its language is provided by taxonomy, the theory and practise of identifying, describing and classifying organisms [1]. Traditionally, taxonomy relied on morphological characters, yet, given the limitations of morphology-based methods in e.g. distinguishing between cryptic species or in interpreting intra-specific variation, an integrative approach of taxonomy has been proposed [2]. A review of the literature, however, revealed a lack of consensus on what methodologies should be included in integrative taxonomy and on how different characters should be weighthed against each other [3]. Schlick-Steiner et al. [1] suggested the use of at least three independent datasets: morphology, nuclear DNA and supporting evidence from one additional discipline. Disciplines that have earned their merits in systematic studies include fields as diverse as mating trails [4–5], bioacoustics [6–7], electric signals [8], cuticular chemistry [9], cytogenetics [10] and ecological niche modeling [11–12].

As species interact with one another, their evolutionary trajectories often run in parallel. Hence, systematic knowledge of one group can provide additional information on a group with which it interacts. Nevertheless, the number of systematic studies that use knowledge of symbionts is limited (but see e.g. [13–14]). Few biological interactions are as intimate as parasitism [15]. Given the co-evolutionary arms race that may occur between a parasite and its host, parasites [16] and parasitoids [17] are often highly species-rich and species-specific. Hence, the distribution of parasites provides additional information about the systematics of their hosts [18–19]. An additional advantage of using parasites relates to their often shorter generation time leading to potentially higher genetic and/or morphological differentiation than observed in their hosts. Therefore, species-specific parasites can provide a magnifying glass that can be used to resolve their hosts’ taxonomy [20]. This is especially promising for hosts belonging to recently or rapidly formed radiations, the taxonomy of which is often difficult to unravel. For such taxa, an integrative approach is almost essential as different datasets often reflect alternative evolutionary scenarios [1].

The cichlid flocks of the East African Great Lakes form the most spectacular vertebrate radiations [21]. Diversification in Great Lake cichlids often occurred in rapid cladogenetic events [22–23], which hampers phylogenetic reconstruction [24–25]. Moreover, the high degree of morphological convergence in species-rich communities [26] also provides a challenge to morphology-based higher order classifications [27]. Lake Tanganyika is, with an estimated age of 9–12 million years [28], the oldest of the East African Great Lakes. Its 250 endemic cichlids form the morphologically, behaviourally and phylogenetically most diverse lacustrine cichlid fauna worldwide, although not the most species-rich [29]. Compared to the radiations of Lake Victoria and Malawi, the systematics of Lake Tanganyika cichlids is relatively well known, yet many problems remain, not in the least at the generic level [27].

In this study, we focus on two genera of Lake Tanganyika cichlids: *Simochromis* Boulenger, 1898 and *Pseudosimochromis* Nelissen, 1977, which belong to the endemic Lake Tanganyika tribe Tropheini. Tropheini are a moderately species-rich and ecologically diverse lineage of Lake Tanganyika cichlids containing 24 valid species in seven genera [30–31]. The tribe has a unique phylogenetic position as it is the sister taxon of the megadiverse assemblage that contains the haplochromine radiations of Lake Victoria and Malawi [32]. Many tropheine species are popular model organisms in evolutionary research [33–35] and species belonging to *Simochromis* have been used as models in ethology [36–38]. Problems, however, remain in species delineation and a nuclear phylogeny [39] even showed *Simochromis* to be paraphyletic (see Historical account).

We aim at reassessing the taxonomy and interrelationships of the species currently classified under *Simochromis* and *Pseudosimochromis* following an integrative approach. For this, we combine morphometric, molecular and parasitological data and compare them with previously published phylogenetic reconstructions [39]. For the parasitological approach, we target monogenean flatworms, which are commonly used to improve the understanding of their host’s biogeography, phylogeny and taxonomy [18–19,40–47]. Monogeneans have a simple one-host lifecycle, high species diversity and a relatively high host-specificity. These traits make them suitable markers to investigate biodiversity and speciation in groups of closely related fishes [48]. The most species-rich genus parasitizing African cichlids is *Cichlidogyrus* Paperna, 1960 [49]. This monogenean genus is often considered to belong to the Ancyrocephalidae, although numerous studies suggest that Ancyrocephalidae does not form a monophyletic group and that its representatives should be considered members of Dactylogyridae [50–53]. Representatives of *Cichlidogyrus* are common on Lake Tanganyika cichlids [35] and 16 species have already been described [44,54–57], six of which infect tropheine cichlids [55,57]. Although no *Cichlidogyrus* species were hitherto formally identified from *Simochromis* and *Pseudosimochromis*, three representatives of the monogenean *Gyrodactylus* von Nordmann, 1832 have been described from Zambian *S*. *diagramma* [58]. We will describe the *Cichlidogyrus* fauna infecting *Simochromis* and *Pseudosimochromis* and compare this fauna with that found on related tropheine genera [39,55,57]. By characterising the morphology and host range of *Cichlidogyrus* species, we aim to shed extra light on the interrelationships of *Simochromis* and *Pseudosimochromis* through an additional line of evidence.

### Historical account

*Simochromis* currently contains five nominal species [31]: *S*. *diagramma* (Günther, 1894), with junior synomym: *Tilapia adolfi* (Steindachner, 1909), *S*. *babaulti* Pellegrin, 1927, *S*. *marginatus* Poll, 1956, *S*. *margaretae* Axelrod & Harrison, 1978 and *S*. *pleurospilus* Nelissen, 1978. *Pseudosimochromis curvifrons* (Poll, 1942) was originally described as a *Simochromis* and *Interochromis loocki* (Poll, 1949) and *Limnotilapia dardennii* (Boulenger, 1899) at some point also belonged to this genus. Both *Simochromis* and *Pseudosimochromis* browse filamentous algae at the lake’s rocky shores although the species differ in their tolerance to sediment [59].

Boulenger [60] erected *Simochromis* together with eight other Lake Tanganyika cichlid genera and with *S*. *diagramma* as the only species. The description was very brief, with the single row of lateral conical teeth given as the sole character separating it from *Tilapia*. Later the same year, Boulenger [61] provided a more detailed genus description. *Simochromis babaulti* was described based on a single specimen from Uvira. Its colour pattern, less steep head profile, larger mouth and lower counts for teeth rows and gill rakers separated this species from its congener [62]. *Pseudosimochromis curvifrons* was originally described as a *Simochromis* [63] but the extreme curvature of the head inspired Nelissen [64] to place it into a separate genus. Five specimens from the Ubwari peninsula were used for the description of *S*. *marginatus* and Poll listed the number of dorsal soft rays, the colour pattern, the higher body, shorter mouth and larger eye as characters to distinguish this species from *S*. *babaulti* [65].

Although there is little doubt concerning the validity of the species listed above, confusion exists on two species described almost simultaneously in 1977 and 1978: *S*. *margaretae* and *S*. *pleurospilus*. Nelissen [66] described the latter based on an observation of P. Brichard that in the southern end of the lake *S*. *babaulti* occurs sympatrically with a highly similar species. Although some morphometric differences were found between *S*. *babaulti* and *S*. *pleurospilus*, all of them overlapped. Hence, the difference in colour pattern, especially the presence of lateral rows of red dots on the flanks in *S*. *pleurospilus* but absent in *S*. *babaulti*, was used to separate the species [66]. Konings [67] already suggested that *S*. *pleurospilus* could represent a southern morph of *S*. *babaulti* and observations along the western shore revealed populations with colour patterns intermediate between those of the typical *S*. *babaulti* and *S*. *pleurospilus* [68]. Moreover, recent molecular results also did not support the monophyly of both species [39]. *Simochromis margaretae* was described on four specimens from Kigoma that were compared with specimens of all congeners and with *P*. *curvifrons*, all of which, except for *S*. *pleurospilus*, were collected at the same locality [69]. Since then, *S*. *margaretae* has never been observed again, despite intensive sampling. This led Konings [67] to speculate that *S*. *margaretae* could be a junior synonym of *S*. *marginatus*.

Besides on the species level, problems also exist on the generic level. Although a cladistic approach showed *Simochromis* to be monophyletic [70], recent nuclear molecular results tell a different story [39]. In an AFLP-based phylogeny of the Tropheini, *Simochromis* was resolved as paraphyletic with *S*. *diagramma* being a separate clade and with *S*. *babaulti*, *S*. *pleurospilus*, *S*. *marginatus* and *P*. *curvifrons* (the latter being sister to the three previous ones) forming a monophyletic clade within the “sediment dwellers” [39]. This group also includes *‘Ctenochromis’ horei* (Günther, 1894), ‘*Gnathochromis’ pfefferi* (Boulenger, 1898) and *Limnotilapia dardennii*.

## Materials and Methods

### Host morphology

In total, 114 specimens, including type material for all nominal species belonging to *Simochromis* and *Pseudosimochromis*, were examined using traditional morphometric techniques. Most specimens belong to the collections of the Royal Museum for Central Africa (Tervuren, Belgium) (RMCA) whereas additional material originates from the Royal Belgian Institute for Natural Sciences (Brussels, Belgium) (RBINS), the Natural History Museum (London, United Kingdom) (BMNH), the Muséum National d’Histoire Naturelle (Paris, France) (MNHN) and the South African Institute for Aquatic Biodiversity (Grahamstown, Republic of South Africa) (SAIAB) (see appendix 1). Eight additional type specimens from the Naturhistorisches Museum (Vienna, Austria) (NMW) were studied on site; however, as they were examined by a different person, they were not included in the morphometric analysis.

Eighteen meristics and 21 measurements were collected. For the meristics, these are: **ASp**: the number of anal spines, **ASR**: the number of anal soft rays, **DSp**: the number of dorsal spines, **DSR**: the number of dorsal soft rays, **Pect**: the number of pectoral rays, **ULL**: the number of pored scales in the upper lateral line, **MLL**: the number of scales in the ‘middle’ lateral line (i.e. the part of the upper lateral line separated from the lower lateral line by only one scale row instead of two), **LLL**: the number of scales in the lower lateral line (when a minute lateral line scale, shorter than half of the previous scale, was present before the articulation of the caudal fin, this was counted as 0.5), **LongL**: the number of scales in the longitudinal line, **CP**: the number or scales around the caudal peduncle, **IOS**: inter-orbital scales, i.e. the smallest number of scales between the eyes, **GRL**: the number of gill rakers on the lower part of the first gill arch (not including the middle gill raker), **GRU**: the number of gill rakers on the upper part of the first gill arch (not including the middle gill raker), **BTU**: the total number of bicuspid teeth in the first row of the upper jaw, **BTL**: the total number of bicuspid teeth in the first row of the lower jaw, **LatT**: the total number of lateral (non bicuspid) teeth on both sides of the upper Jaw, **AV**: the number of abdominal vertebrae and **CV**: the number of caudal vertebrae, the first caudal vertebra is the one to which the first caudal pterygiophore is pointed [71]. X rays were made using the Visix equipment (Medex Loncin SA), which includes a Gem X 160 X ray generator and a high resolution digital X ray detector, Dereo HR1. For the number of lower gill rakers, care was taken to count the reduced gill rakers (sensu [71]) as well. Sometimes, these could only be observed by the different refraction of the light when a concentrated shaft of light was pointed to the anteriormost part of the lower branchial arch [72]. Although not treated separately for the exploratory analyses, reduced and non-reduced gill rakers were recorded separately and listed as such in the character tables.

The 21 measurements are: **TL**: total length (not used in the analyses), **SL**: standard length, **LaD**: lacrimal bone depth, **SnL**: snouth length, **LJL**: lower jaw length, **PPL**: premaxillary processus length, **ED**: eye diameter, **IOW**: inter-orbital width, **MW**: mouth width (measured at the posterior teeth of the upper jaw), **HL**: head length, **BD**: body depth, **PeL**: the length of the longest pectoral ray, **ASL**: the length of the third anal spine, **DFB**: dorsal fin base length, **AFB**: anal fin base length, **PrD**: pre-dorsal distance, **PrP**: pre-pectoral distance, **PrV**: pre-ventral distance, **PrA**: pre-anal distance, **CPL**: caudal peduncle length, **CPD**: caudal peduncle depth. All measurements were taken with a dial calliper up to 0.1 mm. Special care was taken that all of them (except for those from the caudal peduncle) were measured between well-defined bony points. All lateral measurements and meristics were taken on the left side of the body. For the *Simochromis pleurospilus* allotype, the right side of the body was used for scale counts. For the paratype of *S*. *margaretae* for which the lower jaw was missing, the teeth count on the lower jaw was obtained from the original description [69]. For one *S*. *babaulti* specimen, the gill arches had been dissected and the number of lower gill rakers was treated as a missing value. The shape of gill rakers and frontal teeth, the latter based on Yamaoka [59], as well as colour patterns of preserved specimens were qualitatively recorded. Measurments and meristics are given as supporting information.

Meristics and measurements were explored separately using Principal Component Analysis (PCA). This was done on the correlation matrix of the raw meristics and on the covariance matrix of the log-transformed measurements. For the PCA conducted on the log-transformed measurements, the first PC is interpreted as describing growth [73]. Variable loadings are given as coefficients. Pair-wise Mann-Whitney U-tests were conducted on the percentages of the linear measurements as well as on the meristics. Percentages of measurements were expressed with respect to head (HL) and standard length (SL), for measurements on the head and the rest of the body respectively. As these measurements as well as the teeth counts could contain allometric variation, these tests were conducted on subsets of similar size class specimens (p>0.1 for SL). As there is discussion regarding the status of *S*. *pleurospilus* versus that of *S*. *babaulti*, different populations of *S*. *babaulti* were examined separately and compared with *S*. *pleurospilus*. For this, *S*. *babaulti* specimens were grouped according to their geographic origin: the north (including the type locality: Uvira, Kavimvira, Bujumbura, n = 6), the northeast (Segunga, Kalela, Kabwe, n = 8), the east (Mpimbwe Hills, Kasinde, Karema, ‘midway between Ikola and Mkangasi’, Msamba Bay Kalia, Ulwile Island and Kapele, n = 22) and the west (Mukamba and Kyanza, n = 11). We considered all specimens originating from the extreme southeastern end of the lake (Chaitika, Cap Kabeyeye, Cap Nundo, Kama Bay, n = 13) as belonging to *S*. *pleurospilus*. Bonferroni correction was performed for the Mann-Whitney U tests on individual variables.

### Host genetics: barcoding

For 30 specimens (*S*. *diagramma* (n = 5), *S*. *babaulti* (n = 16), *S*. *marginatus* (n = 4), *S*. *pleurospilus* (n = 3) and *P*. *curvifrons* (n = 2)) encompassing all the nominal taxa except *S*. *margaretae*, the first subunit of the cytochrome *c* oxidase gene (COI) was sequenced following [74]. As these species do not form a monophylum, specimens from all other “sediment dwelling” tropheine species were included in the barcoding analysis: *L*. *dardennii* (n = 3), ‘*C*.’ *horei* (n = 3) and ‘*G*.’ *pfefferi* (n = 3). Sequences are available at NCBI GenBank under accession numbers KP336420-KP336458. Sequence analysis was carried out in MEGA v.5 [75]. Model selection performed using the same software indicated the Kimura-2-parameter [76] + Γ model (with gamma-shape parameter = 0.14) as optimal model of molecular evolution, based on the Bayesian information criterion. Pairwise genetic distances were calculated in MEGA according to this model.

### Parasite morphology

Host cichlid fish were either retrieved from the RMCA collections, collected on site using gill nets or purchased from local fishermen and subsequently deposited in the RMCA collections. They were identified to species level on site by Christian Sturmbauer (Karl-Franzens University of Graz, Austria) or Donatien Muzumani Risasi (Centre de Recherche en Hydrobiologie-Uvira, DRC) and ex situ by the authors. Newly collected fish were kept alive in aerated tanks until they were sacrificed by severing the spinal cord or with an overdose of MS-222. The entire fish or the right branchial arches were stored in pure ethanol. Samples were collected under research permit no. 2007-258-CC-2006-151 from the Tanzania Commission for Science and Technology (COSTECH), under mission statement no. 013/MNRST/CRHU/2010 from the Congolese Ministère de la Recherche Scientifique et Technologique—CRH-Uvira and with permits from the Department of Fisheries, Zambian Ministry of Agriculture and Co-operatives. Gills were inspected for parasites under a Wild M5 (field), Olympus SZX12 or Wild M8 (laboratory) stereomicroscope. Monogenea were removed with a dissection needle. They were mounted on a slide under a cover-slip and fixed using ammonium picrate glycerine [77].

**Fig 1:**
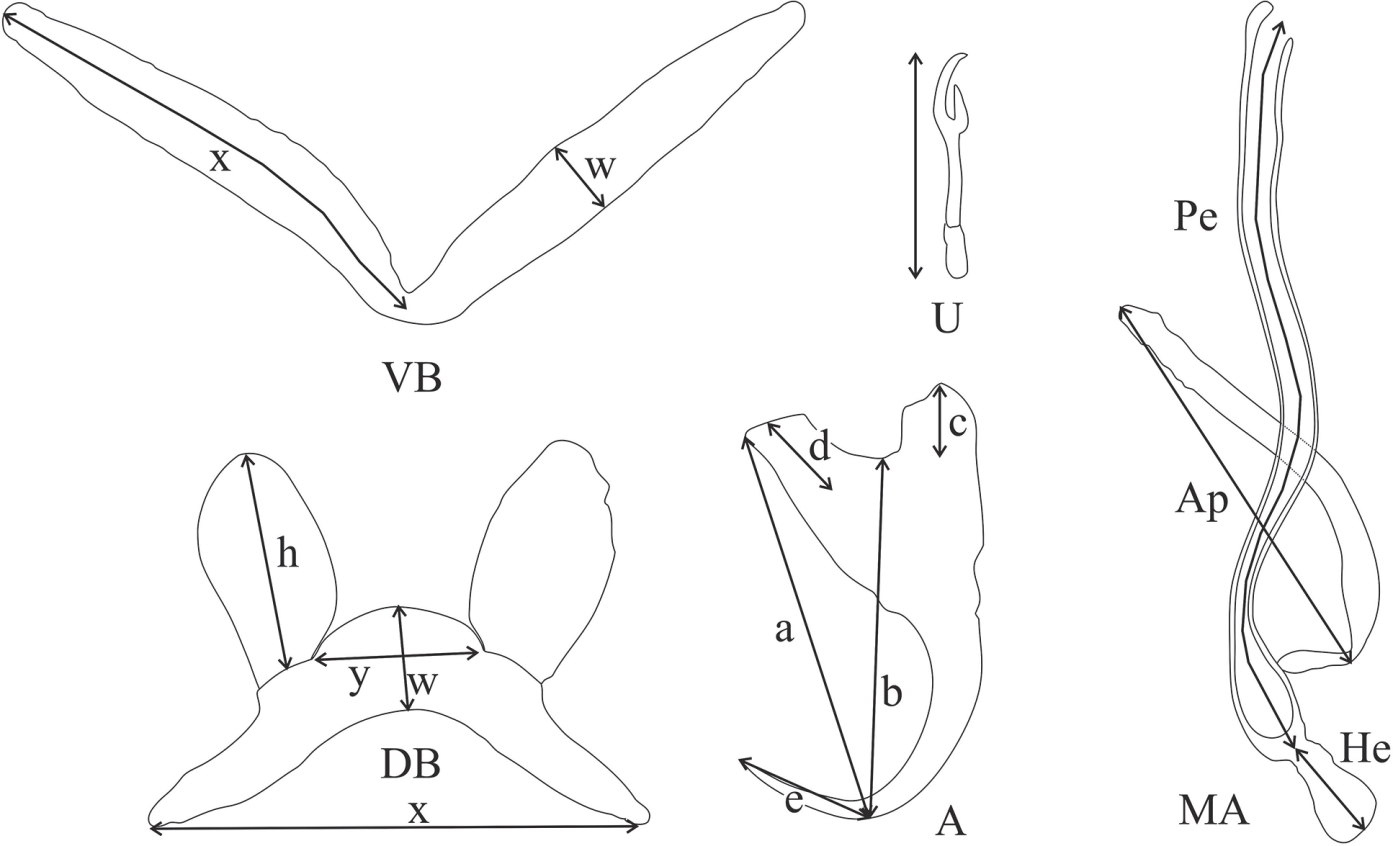
Measurements used to study the new *Cichlidogyrus* species. (DB) dorsal transverse bar: (h) length of dorsal bar auricle, (w) dorsal bar maximum width, (x) dorsal bar total length, (y) distance between auricles. (A) anchor: (a) anchor total length, (b) anchor blade length, (c) anchor shaft length, (d) anchor guard length, (e) anchor point length (both dorsal and ventral anchors were examined). (MA) male apparatus: (Ap) accessory piece length, (Pe) penis total length, (He) heel length. (U) uncinuli length. (VB) ventral transverse bar: (w) ventral bar maximum width, (x) length of one ventral bar branch.

Pictures and measurements of the hard parts of haptor and male copulatory apparatus (MA) were taken based on [78] using a Leica DM2500 microscope at a magnification of 1000x (oil immersion, 10x ocular) with the software LAS v.3.6 and a DFC 425 Leica camera. The numbering of haptoral parts was adopted from ICOPA IV [79]; terminology follows [80] (i.e. “uncinuli” for marginal hooks) and the measurements taken are shown in Fig 1. Measurements are in micrometers and presented as the average + the standard deviation, with the range in parentheses and the number of measured specimens in superscript. Type material was deposited in the invertebrate collection of the RMCA, in the MNHN, and in the Iziko South African Museum (Cape Town, Republic of South Africa) (SAMCTA). When the slide containing the holotype of a species also contained other specimens, holotypes were individually marked. Symbiotypes [81] and host vouchers [82] were deposited in the RMCA.

### Nomenclatural acts

The electronic edition of this article conforms to the requirements of the amended International Code of Zoological Nomenclature, and hence the new names contained herein are available under that Code from the electronic edition of this article. This published work and the nomenclatural acts it contains have been registered in ZooBank, the online registration system for the ICZN. The ZooBank LSIDs (Life Science Identifiers) can be resolved and the associated information viewed through any standard web browser by appending the LSID to the prefix "http://zoobank.org/". The LSID for this publication is: urn:lsid:zoobank.org:pub:765EC2C4-3413-4379-B24A-E2D121BFC204. The electronic edition of this work was published in a journal with an ISSN, and has been archived and is available from the following digital repositories: PubMed Central, LOCKSS.

## Results

### Host morphology: inter-specific analysis of *Simochromis* and *Pseudosimochromis*

#### Meristics

**Fig 2:**
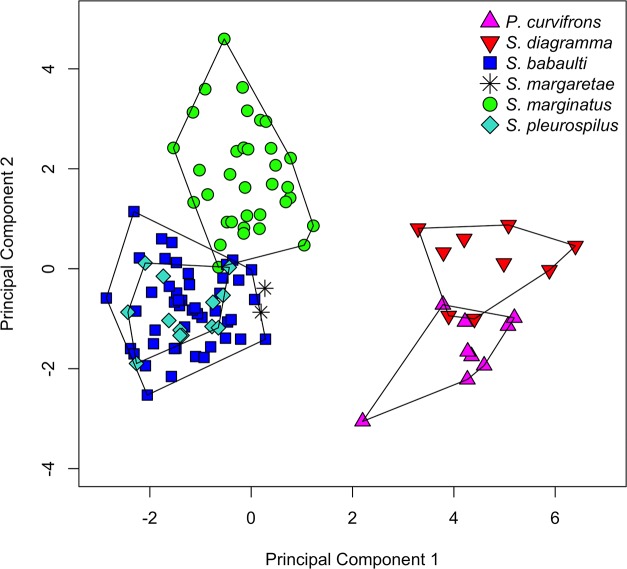
PCA on meristics of the inter-specific analysis. PC2 vs. PC1 of a PCA on 16 meristics from 114 specimens belonging to the six *Simochromis* and *Pseudosimochromis* species.

The 16 meristics that contained variation for all 144 specimens were analysed using PCA (Table 1). The number of scales around the caudal peduncle and the number of anal spines were constant (16, resp. 3) and thus omitted from the analysis. A scatter-plot of the second versus the first PC is presented in Fig 2. The first PC, explaining 28.39% of the variance, separated *S*. *diagramma* and *P*. *curvifrons* from *S*. *babaulti*, *S*. *margaretae*, *S*. *marginatus* and *S*. *pleurospilus*, further called the ‘small’ *Simochromis*. For this axis, values for both *S*. *margaretae* paratypes were higher than those for all but one of the *S*. *babaulti* and *S*. *pleurospilus* specimens. The most important variables in this PC were the number of anal soft rays (ASR), abdominal vertebrae (AV) and lower gill rakers (LGR). The second PC, explaining 14.21% of the variance, allowed for an almost complete separation between *S*. *diagramma* and *P*. *curvifrons*. This axis also separated *S*. *marginatus* from *S*. *margaretae* and, albeit with some overlap, from *S*. *pleurospilus* and *S*. *babaulti*. The main contributors to this axis were the number of bicuspid teeth on the upper and lower jaw (BTU, BTL) and the number of dorsal soft rays (DSR). A separation between *P*. *curvifrons* and *S*. *diagramma* was also obtained by axis 3 and 4 whereas axis 3 and 5 again separated *S*. *marginatus* from *S*. *margaretae* (results not shown). None of the PC could distinguish *S*. *pleurospilus* from *S*. *babaulti* and these species will be hence investigated later (see *S*. *babaulti* versus *S*. *pleurospilus*).

**Table 1:** PCA on meristics of the inter-specific analysis.

	PC1	PC2
Variance (%)	28.391	14.207
ASR	0.4021	-0.1524
DSp	0.2020	0.3169
DSR	0.1386	-0.3505
Pect	0.2214	0.0052
ULL	-0.0422	-0.1119
MLL	-0.2181	-0.2666
LLL	0.3175	0.1874
LongL	0.3041	0.0758
IOS	0.2699	-0.1208
GRU	0.0120	0.3265
GRL	0.3548	0.1434
BTU	-0.1316	0.5220
BTL	-0.2443	0.4458
LatT	0.1923	0.0233
AV	0.3896	0.1234
CV	-0.1483	-0.0144

Loadings and explained variance of the first four PC of a PCA conducted on the correlation matrix of 16 meristics taken on 114 specimens.

A summary of the meristics for all species examined and the results of the Mann-Whitney U tests are presented in Table 2. Here, values for *S*. *babaulti* and *S*. *pleurospilus* were grouped together given that they could not be separated in the PCA. Although 56 comparisons of meristics were found to differ significantly between the species by using Mann-Whitney U tests, very few allowed for the separation of species. Indeed, although *S*. *diagramma* and *P*. *curvifrons* and the ‘small’ *Simochromis* species formed two distinct groups in the exploratory analysis, all meristics overlapped between them. In fact, only two meristics, the number of inter-orbital scales (IOS) and the number of lower biscuspid teeth (BTL), could be used to separate *P*. *curvifrons* from the ‘small’ *Simochromis* species (4–6 vs 2–3 for IOS and 20–22 vs. 24–40 for BTL). Between *S*. *diagramma* and the ‘small’ *Simochromis* species, all meristics overlapped. For one meristic: the number of lower lateral line scales (LLL) values for *S*. *diagramma* were significantly higher than for all the ‘small’ *Simochromis* species (12–14.5 vs. 8–13). Moreover, *S*. *diagramma* and *P*. *curvifrons* had eight or nine soft anal fin rays (ASR) whereas on all but one of the *S*. *babaulti*, *S*. *pleurospilus* and *S*. *marginatus* specimens seven or six were counted. The two *S*. *margaretae* paratypes had seven and eight soft anal rays respectively. The number of bicuspid teeth in the upper and lower jaw could be used to distinguish *P*. *curvifrons* from *S*. *diagramma* (21–28 vs. 31–39 for BTU and 20–22 vs. 23–30 for BTL).

**Table 2:** Summary of meristics of the different *Simochromis* and *Pseudosimochromis* species.

	*S*. *diagramma*	*P*. *curvifrons*	*S*. *babaulti—S*. *pleurospilus*	*S*. *marginatus*	*S*. *mae*	M-W U
N	9	9	60	34	2	
DSp, DSR	XVI,10(1); XVII,9(1); XVII,11(2); XVIII,9(1); XVIII,10(2); XVIII,11(2)	XVI,10(1); XVII,9(2); XVII,10(1); XVIII,9(4); XVIII,10(1)	XVI,9(4); XVI,10(18); XVI,8(3); XVII,9(25); XVII,10(2); XVIII,9(1)	XVI,9(5); XVII,8(3); XVII,9(14); XVIII,8(10); XVIII,8(6)	XVII, 9	d,c,m>b (DSp) d>b; c,b>m (DSR)
Asp, ASR	III, 8(8); III, 9(1)	III, 8(7); III, 9(2)	III, 7(60)	III, 6(1); III, 7(32); III, 8(1)	III, 7; III, 8	d,c,me>b,m (ASR)
ULL: /—MLL	22-0(2); 23-0(4); 24-0(1); 25-0(2)	20-0(1); 21-0(1); 21-2(1); 22-0(3); 23-1(1); 24-0(2)	20-3(1); 21-0(4); 21-1(3); 21-2(5); 22-0(8); 22-1(20); 22-2(5); 22-3(3); 23-0(4); 23-1(4); 23-2(2); 23-3(1)	21-0(1); 22-0(16); 22-2(2); 22-3(1); 23-0(9); 23-1(3); 23-2(1); 24-0(1)	22–0; 23–0	b>d,m (MLL)
LLL	12(2); 13(3); 14(2); 14.5(2)	9(1); 10(2); 10.5(1); 11(2); 12(2); 13(1)	8(7); 8.5(1); 9(20); 9.5(3); 10(19); 10.5(2); 11(6); 12(1); 12.5(1)	8(1); 9(3); 10(9); 10.5(3); 11(10); 11.5(4); 12(3); 13(1)	10; 11	d>c,b,m,me d,c,m>b
LongL	33(5); 33.5(1); 34(1); 34.5(1)	32(4); 31.5(1); 33(4)	30.5(2); 31(17); 31.5(5); 32(34); 33(2); 34(1)	31(2); 31.5(4); 32(23); 32.5(3); 33(2)	31; 32	d>b,m
Pect	15(1); 16(8)	15(8); 16(1)	14(1); 15(57); 16(2)	14(1); 15(31); 16(2)	13; 14	d>c,b,m; b>me
LGR-1-UGR	10-1-2(1); 11-1-2(1); 11-1-3(1); 11-1-4(1); 12-1-2(1); 12-1-3(1); 12-1-4(1); 13-1-3(2)	10-1-3(4); 10-1-4(1); 11-1-4(1); 12-1-3(2); 12-1-4(1)	4-4-1-3(1); 1-5-1-3(1); 2-5-1-3(3); 3-5-1-2(1); 3-5-1-3(21); 3-5-1-4(1); 4-5-1-2(1); 4-5-1-3(4); 4-5-1-4(1); 5-5-1-3(1); 2-6-1-3(7); 2-6-1-4(1); 3-6-1-3(9); 2-6-1-4(1); 4-6-1-3(1); 2-7-1-3(4); 1-8-1-4(1); 2-8-1-3(1)*	4-5-1-3(6); 4-5-1-4(3); 5-5-1-3(4); 5-5-1-5(1); 3-6-1-3(4); 3-6-1-4(2); 3-6-1-5(1); 4-6-1-4(5); 4-6-1-5(1); 5-6-1-3(2); 5-6-1-4(2); 2-7-1-4(1); 3-7-1-4(2)	2-7-1-2	d,c>m>b (LGR) m>b; b>me (UGR)
BTU	31(2); 34(3); 35(1); 36(1); 39(2)	21(1); 22(1); 26(3); 27(2); 28(2)	26(1); 28(3); 29(4); 30(7); 31(6); 32(11); 33(6); 34(11); 35(3); 37(2); 38(5); 43(1)	34(5); 35(4); 36(5); 37(5); 38(4); 39(3); 40(3); 41(3); 42(2)	32; 36	m>b>c; d>c
BTL	23(1); 24(2); 25(1); 26(3); 29(1); 30(1)	20(3); 21(2); 22(4)	24(3); 25(1); 26(5); 27(2); 28(7); 29(3); 30(11); 31(7); 32(6); 33(4); 34(9); 35(1); 37(1)	25(1); 26(2); 30(3); 31(2); 32(4); 34(9); 35(1); 36(3); 38(3); 39(1); 40(5)	30; **24**	m>b>d>c
LatT	12(2); 15(4); 16(1); 17(1); 18	11(1); 12(1); 13(2); 15(1); 16(1); 17(1); 18(2)	4(1); 6(2); 7(1); 8(4); 9(5); 10(7); 11(5); 12(9); 13(6); 14(7); 15(4); 16(5); 17(3); 18(1)	8(1); 9(1); 10(4); 11(5); 12(5); 13(6); 14(7); 15(2); 16(1); 17(1); 18(1)	16; 20	
IOS	2(4); 3(3); 4(2)	4(3); 5(5); 6(1)	2(46); 3(14)	2(20); 3(14)	2; 3	c>d,b,m
AV + CV	14+17(1); 15+17(5); 15+18(2); 16+17(1)	14+16(1); 14+17(1); 15+15(2); 15+16(1); 15+17(4)	13+16(2); 13+17(17); 13+18(18); 14+16(1); 14+17(19); 14+18(3)	13+18(1); 14+16(1); 14+ 17(32)	14+16	b>m>c; m>me (CV) c,d>m>b (AV)

Values for *S*. *pleurospilus* and *S*. *babaulti* are grouped together. Mann-Whitney U tests are summarised with d: *S*. *diagramma*, c: *P*. *curvifrons*, b: *S*. *babaulti* and *S*. *pleurospilus*, m: *S*. *marginatus* and me: *S*. *margaretae* (*S*. *mae*). ULL is the sum of the two values, reduced and non-reduced gill rakers are listed separately for *S*. *babaulti*, *S*. *pleurospilus*, *S*. *marginatus* and *S*. *margaretae*.

**Fig 3:**
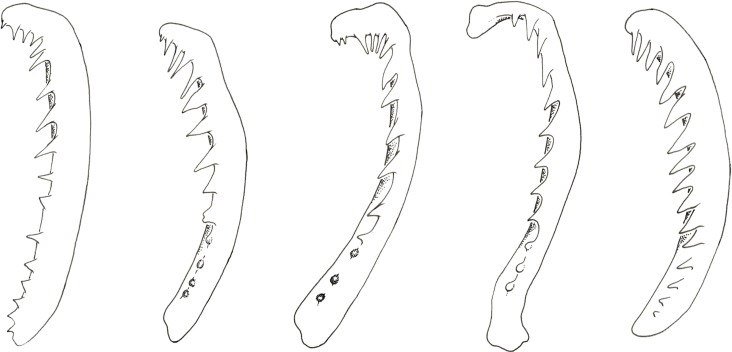
Gill arches of *Simochromis* and *Pseudosimochromis* species. From left to right: *P*. *curvifrons*, *S*. *babaulti*, *S*. *marginatus*, *S*. *margaretae* and *S*. *diagramma*. Specimens illustrated are listed in Appendix.

When the shape of the gill rakers was taken into account, the two main groups identified by PC1 could be separated. Indeed, whereas in *S*. *marginatus*, *S*. *babaulti*, *S*. *pleurospilus* and *S*. *margaretae* there was a clear transition between the blunt reduced and the sharp non-reduced gill rakers, this was not the case in *S*. *diagramma* or in *P*. *curvifrons* (Fig 3). In the latter species, reduced gill rakers were never observed and sharp gill rakers were present up until the anteriormost part of the first lower gill arch. In *S*. *babaulti* and *S*. *pleurospilus* the difference between the non-reduced and the reduced gill rakers was the most striking and the reduced gill rakers could only be observed by their different refraction of light [72]. The anteriormost of the non-reduced gill rakers, however, was always visible as protruding out of the fleshy tissue. A similar situation was encountered in *S*. *marginatus* although, in this species, some of the reduced gill rakers protruded slightly, but were still blunt. In the two *S*. *margaretae* paratypes, the two anteriormost lower gill rakers differed from the seven other gill rakers both by their smaller size and by their blunt shape. Yet, they were more developed than in the other ‘small’ *Simochromis* species and clearly visible. In *S*. *diagramma*, the anteriormost lower gill rakers were small but there was a gradual transition between the long villiform lower gill rakers dorsally and the short gill rakers ventrally. Also, all gill rakers clearly protruded from the fleshy tissue. In *P*. *curvifrons*, a similar gradual reduction in size was encountered although with less difference in shape as all gill rakers were relatively short. Hence, the number of sharp non-reduced lower gill rakers (4–8 vs. 10–13) separated the small *Simochromis* species from *S*. *diagramma* and *P*. *curvifrons*.

As was the case for the gill rakers, the shape and arrangement of the frontal teeth also differed between the species. In *P*. *curvifrons*, frontal teeth were densely set and had a nearly symmetrical shape, with the medial and lateral cusp being nearly equally long and with only a small notch between them. A similar situation was observed in *S*. *margaretae* although here the notch between the cusps was relatively deep. In *S*. *babaulti*, *S*. *pleurospilus* and *S*. *marginatus* teeth were also densely set. In these species, the two cusps were asymmetrical with the median cusp being larger than the lateral. In *S*. *diagramma* teeth were not as densely set and there was a gap between individual teeth. In this species, the medial cusp, which extended over the gap between the teeth, was much larger than the lateral cusp (see [59]). Another criterion mentioned by Yamaoka [59] to separate *Simochromis* from *Pseudosimochromis* was the distance between the outer row of bicuspid teeth and the first row of tricuspid teeth. While this distance was small in *P*. *curvifrons*, a relatively large distance (i.e. larger than the distance between the consecutive rows of tricuspid teeth) was observed in *S*. *diagramma*, *S*. *babaulti*, *S*. *pleurospilus* and *S*. *marginatus*. In *S*. *margaretae*, however, this distance was also small.

Finally, in most *S*. *babaulti* and *S*. *pleurospilus* specimens, the upper lateral line ran over two adjacent longitudinal scale rows. Hence, the anterior section of the upper lateral line was separated from the lower lateral line by two transverse scales and the posterior section by only one transverse scale. As the upper lateral line was always confluent, the pore on the posterior most scale of the anterior section of the upper lateral line was bended down to connect to the pore on the first scale of the posterior section. This posterior section was here named the ‘middle’ lateral line (MLL). When no such ‘middle’ lateral line was observed, the pore of the posteriormost scale of the upper lateral line was also bended down in *S*. *babaulti* and *S*. *pleurospilus*. In *S*. *marginatus*, *P*. *curvifrons* and *S*. *margaretae*, a ‘middle’ lateral line was sometimes present. For the latter species, this was not observed on the paratypes examined, although it was visible in the illustration of the holotype [69]. In the cases where in these three species no ‘middle’ lateral line was present, the last pore of the upper later line was, however, not bended down. In *S*. *diagramma*, a ‘middle’ lateral line was never observed and the pore on the last scale of the upper lateral line was always straight.

#### Measurements

**Fig 4:**
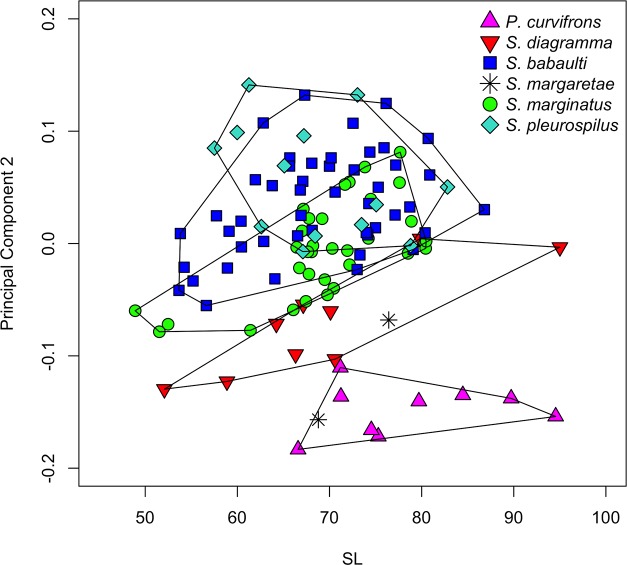
PCA on measurements of the inter-specific analysis. PC2 of a PCA on the 20 measurements from 114 specimens belonging to the six *Simochromis* and *Pseudosimochromis* species, plotted vs. SL.

A PCA was performed on 20 log-transformed measurements of 114 specimens (Table 3). For the paratype of *S*. *margaretae* of which the lower jaw wa s missing, the lower jaw length was interpolated by assuming that its length relative to the head length is identical to that of the other paratype. The first PC, explaining 82.60% of the variance, explained growth and was not investigated [73]. Although the second principal component included an allometric effect, with lower within-group values for smaller specimens than for larger, it did separate different groups (Fig 4). *Pseudosimochromis curvifrons* specimens had the smallest values for PC2, *S*. *diagramma* was intermediate and *S*. *marginatus*, *S*. *babaulti* and *S*. *pleurospilus* had the highest values. Within the latter group, values for *S*. *babaulti* and *S*. *pleurospilus* were, on average, higher than those for *S*. *marginatus*. Values for the two *S*. *margaretae* specimens overlapped with those of *S*. *diagramma* and *P*. *curvifrons*. The second PC explained 5.92% of the variance and had the inter-orbital width (IOW) as its main contributor. Some separation was also observed on PC3 to PC5 (not shown). The third PC only allowed for a separation between *P*. *curvifrons* and *S*. *diagramma*. PC4 separated *S*. *margaretae*, with the highest values for this axis, from all other species. Finally, PC5 was the first that separated *S*. *babaulti* and *S*. *pleurospilus* from *S*. *marginatus*, although with a considerable amount of overlap. Even when a subsequent PCA, restricted to the specimens of *S*. *babaulti*, *S*. *pleurospilus* and *S*. *marginatus* was performed, the latter species could only be partially separated (not shown). None of the PCs separated *S*. *babaulti* from *S*. *pleurospilus* and measurements of these species will be investigated later (see *S*. *babaulti* versus *S*. *pleurospilus*).

**Table 3:** PCA on measurements of the inter-specific analysis.

	PC1	PC2	PC3	PC4	PC5
VAR (%)	82.6090	5.9182	2.4578	2.2426	1.7082
SL	0.2014	0.0383	0.0868	0.1341	0.0318
LaD	0.2896	0.1078	0.0133	-0.3102	-0.0260
SnL	0.2775	0.1465	-0.0470	-0.2431	0.0112
LJL	0.2025	0.3823	0.0777	-0.4347	-0.1361
PPL	0.2279	-0.2269	0.1024	-0.4303	-0.1345
ED	0.1484	0.2124	-0.0897	0.0724	0.2817
IOW	0.3244	-0.6834	-0.3457	-0.0446	-0.0669
MW	0.2720	0.3373	-0.2728	0.3122	-0.4225
HL	0.2092	0.1073	-0.0775	-0.1547	0.0222
BD	0.2313	-0.1361	0.0751	-0.0220	0.1890
PeL	0.2377	-0.0667	0.0029	0.0670	0.6384
ASL	0.1332	0.0836	0.2477	0.1187	0.0269
DFB	0.2170	-0.1084	0.2758	0.1063	-0.0432
AFB	0.2243	-0.1103	0.3294	0.1788	-0.3463
PrD	0.2105	0.0817	0.0880	-0.1304	0.1239
PrP	0.1926	0.1728	-0.2777	0.1032	0.0126
PrV	0.1917	0.0969	-0.3588	0.2725	0.1276
PrA	0.1995	0.0541	-0.1118	0.1716	0.0667
CPL	0.1707	0.0570	0.5292	0.2701	0.1584
CPD	0.2201	-0.1564	0.0885	0.2395	-0.2677

Loadings and explained variance of the first five PC of a PCA conducted on the covariance matrix of 20 log-transformed measurements taken on 114 specimens.

The relative measurements of all specimens and the results of the Mann-Whithey U tests are summarised in Table 4. Here as well, values for *S*. *pleurospilus* were lumped with those of *S*. *babaulti*. Forty-six relative measurements differed significantly between the species. In spite of the absence of significant differences between *S*. *margaretae* and the other species, due to the limited number of specimens available, the species’ distinction was obtained directly from the measurements. Indeed, the measurements for the length of the lower jaw (LJL) were smaller and those of the depth of the caudal peduncle (CPD) larger than those measured for all other species. Moreover, while *S*. *margaretae* clustered somewhat with *P*. *curvifrons*, five other measurements; i.e. the lacrimal depth (LaD), snout length (SnL), premaxillary processus length (PPL), eye diameter (ED) and pre-ventral length (PrV) did not overlap between both species. *Simochromis babaulti*, *S*. *pleurospilus* and *S*. *marginatus* could be separated from the other species by their smaller relative inter-orbital with (IOW) (17.3–28.3 vs. 29.1–37.0). The pre-ventral distance separated *P*. *curvifrons* from *S*. *diagramma* (33.6–37.4 vs. 39.2–43.5). Finally, the caudal peduncle was always longer than deep in *S*. *babaulti*, *S*. *pleurospilus* and S. *marginatus* (64.2–97.1%) whereas it was always deeper than long in *S*. *margaretae* (108.4–110.9%).

**Table 4:** Summary of measurements of the different *Simochromis* and *Pseudosimochromis* species.

	*S*. *diagramma*			*P*. *curvifrons*			*S*. *babaulti*			*S*. *marginatus*			*S*. *margaretae*			MWU
N	9			9			60			34			2			
	min	mean	max	min	mean	max	min	mean	max	min	mean	max	min	mean	max	
SL (mm)	52.1	69.4	95.0	66.6	78.6	94.6	53.7	68.7	86.8	48.9	69.7	80.5	68.8	72.6	76.4	
LaD°	20.1	23.0	25.3	23.5	25.5	28.8	19.9	23.0	25.7	19.9	23.0	26.5	19.5	19.8	20.1	c>b,m
SnL°	33.1	38.7	41.6	38.7	40.5	41.9	34.1	38.7	43.6	32.5	39.2	45.2	36.0	36.3	36.6	
LJL°	26.5	30.1	31.2	25.2	27.5	29.9	26.0	31.4	34.3	25.4	30.1	34.9	23.2	23.2	23.2	d>c; b>m>c
PPL°	25.0	28.5	30.8	30.4	32.3	34.3	21.4	26.5	29.1	23.9	26.6	29.9	24.6	25.2	25.8	c>m; c>d >b
ED°	32.2	34.3	35.5	29.7	31.0	32.4	33.5	36.0	39.9	33.1	37.4	42.7	36.4	36.8	37.2	m>b>d>c
IOW°	29.1	31.2	33.7	29.2	33.5	37.0	17.3	22.8	27.5	23.0	25.7	28.3	29.3	31.6	33.9	d,c>m>b
MW°	29.8	34.1	39.7	26.2	29.6	34.2	27.4	33.9	41.0	28.2	32.8	40.9	32.4	33.3	34.1	b,m>c
HL*	30.2	31.5	33.0	28.8	31.2	33.2	29.0	30.9	35.3	28.9	30.7	34.0	31.5	31.7	31.9	
BD*	35.4	37.5	41.5	36.7	39.3	41.8	31.0	35.9	39.5	32.5	37.7	41.0	37.7	37.7	37.8	c,m>b
PectL*	29.5	31.2	33.5	28.8	32.6	36.8	25.5	30.4	34.1	31.2	33.7	36.3	29.4	30.4	31.3	m>d,b
ASp*	13.9	15.6	17.0	12.6	14.3	15.9	13.8	15.9	18.4	13.8	15.3	17.6	16.0	16.6	17.2	b>m
DFB*	56.7	58.8	61.0	60.2	62.4	65.3	52.6	58.5	62.3	54.3	57.7	60.2	58.9	60.8	62.6	c>d,b,m
AFB*	16.2	18.1	19.8	18.4	19.5	21.3	15.6	18.2	20.1	15.6	17.2	18.7	18.2	18.8	19.5	c>b,m
PrD*	34.1	36.0	37.6	34.7	36.6	39.0	32.7	36.4	40.4	33.4	37.0	40.7	36.2	36.8	37.5	
PrP*	31.8	32.6	32.8	28.1	29.7	32.3	28.5	31.1	36.8	28.0	31.0	33.8	31.8	32.4	33.0	d>c
PrV*	39.2	41.5	43.5	33.6	35.6	37.4	34.2	37.5	43.8	35.2	38.9	43.9	40.1	41.6	43.1	d,m>c,b
PrA*	68.1	70.4	71.2	62.9	65.7	68.7	63.4	66.7	70.7	64.6	67.9	70.7	66.7	68.9	71.2	d>m>c,b
CPL*	12.2	13.8	15.5	11.6	14.1	16.1	12.8	14.5	16.1	12.1	14.2	16.7	12.3	12.3	12.4	
CPD*	11.7	12.2	12.9	11.1	12.1	12.9	9.9	11.3	12.8	10.1	11.1	12.0	13.3	13.5	13.7	d>b,m; c>m

Measurements are indicated as percentage of head ° or standard length *, for the 114 specimens studied. Values for *S*. *pleurospilus* are grouped with those of *S*. *babaulti*. Results of Mann-Whitney U tests are summarised with d: *S*. *diagramma*, c: *P*. *curvifrons*, b: *S*. *babaulti* and *S*. *pleurospilus* and m: *S*. *marginatus*.

#### Colour patterns

In about half of the *S*. *babaulti* and *S*. *pleurospilus* specimens, one to three anal ocelli were present, both in males and, albeit less frequently, in females. A dark spot on the anteriormost part of the dorsal fin was observed in almost all specimens. This spot was black and clearly visible in most of the males, whereas it ranged from back to light brown in the females. On specimens from the northern and the northeastern shores, the spot measured about one third of the height of the dorsal fin and was equidistant from its proximal and its distal end. In specimens from all other areas this spot was higher and covered the distal two thirds of the anteriormost part of the dorsal fin. Eight to nine lateral bands were visible on the flanks of most specimens.

On *S*. *marginatus*, a single ocellus was observed in five of the 34 specimens, all of which were males. The dark edge on the dorsal fin, which gave the species its name [65] was visible as a very narrow edge in specimens from the Kavala Islands (Musinwa), from the extreme north (Luhanga) and from the Burton Bay (Lubumba and Kisokwe) whereas specimens caught at the northern and the eastern side of the Ubwari peninsula (Manga, Cap Banza and Ubwari ‘East’ and ‘West’) had a broad dorsal band that covered the distal third of the dorsal fin and that broadened posteriorly. In the specimens from the eastern shore, a broad band was observed on the male whereas a narrow band was present on the two females. Up to seven lateral bands were counted on the dorsal half of the flanks of some specimens, although, on others, none were visible. The two *S*. *margaretae* specimens had a broad band on the distal half of the dorsal fin. Seven vertical bars and two horizontal bands following the lateral lines were visible on the flanks. No ocelli were observed. In *P*. *curvifrons* and in *S*. *diagramma*, lateral bands and anal ocelli were present on some specimens. Dark spots were observed on the membranes between the dorsal spines but a clear band on the dorsal fin was lacking.

### *Simochromis babaulti* versus *S*. *pleurospilus*

#### Meristics

**Fig 5:**
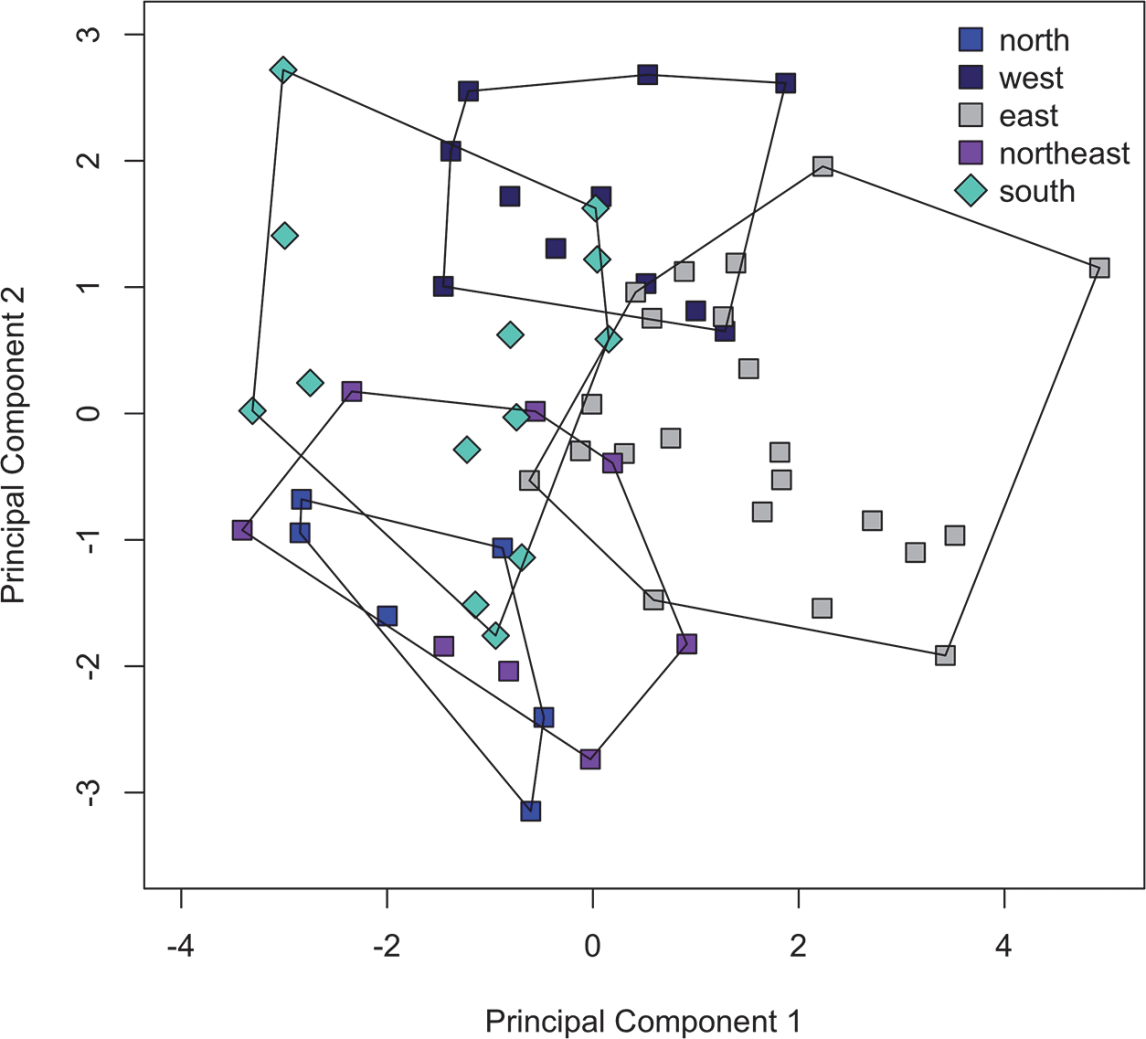
PCA on meristics of *S*. *babaulti* and *S*. *pleurospilus*. PC2 vs. PC1 of a PCA on the 15 meristics for 60 specimens belonging to four geographical groups of *S*. *babaulti* and to *S*. *pleurospilus* (the southern group).

As no separation could be obtained between *S*. *pleurospilus* and *S*. *babaulti* in the previous analyses, a subsequent PCA was performed on the two species separately, split up in geographical units (Table 5). The number of soft anal rays was always seven and this variable was omitted from the analysis. On a scatter-plot of the first versus the second PC four partially overlapping groups were identified (Fig 5): a combined northern and northeastern group, a western group, a southern group (*S*. *pleurospilus*) and an eastern group. The first two groups were separated by PC2, the latter two, incompletely, by PC1. No separation was observed on PC3, whereas PC4 separated northern and northeastern *S*. *babaulti* (not shown). The main contributors for PC1 were the number of lower gill rakes (LGR) and upper lateral line scales (ULL); for PC2, these were the number of dorsal soft rays (DSR), longitudinal line scales (LongL) and caudal vertebrae (CV) and for PC4 these were the number of abdominal vertebrae (AV) and mid-lateral line scales (MLL).

**Table 5:** PCA on meristics of *S*. *babaulti* and *S*. *pleurospilus*.

	PC1	PC2	PC3	PC4
Variance (%)	21.5600	13.4960	11.9570	9.6323
DSp	0.3487	-0.1932	-0.3144	0.0601
DSR	-0.1702	0.4863	0.0638	0.1935
Pect	-0.0918	-0.1769	0.2166	0.3860
ULL	0.4348	0.0231	0.1153	-0.2255
MLL	0.3142	-0.1228	0.1641	-0.4263
LLL	-0.2332	0.3035	-0.0703	0.1341
LongL	0.2406	0.4751	-0.1916	0.1457
IOS	0.2078	-0.0618	0.3064	0.2438
GRU	0.1943	0.1292	0.0065	0.2848
GRL	-0.3577	-0.0827	-0.0106	-0.2308
BTU	0.2273	0.2512	0.3778	-0.1726
BTL	0.0246	0.2484	0.6020	-0.0574
LatT	-0.2892	-0.0766	0.2854	0.0552
AV	0.3071	-0.0890	-0.0186	0.5012
CV	0.0432	0.4422	-0.2955	-0.2388

Loadings and explained variance of the first four PC of a PCA conducted on the correlation matrix of 15 meristics taken on 60 specimens.

Pairwise Mann-Whitney U tests showed that the different geographical groups differed significantly in meristics for 23 pair wise comparisons (Table 6). For all but two of the comparisons between the groups, the northeastern versus the northern and the northeastern versus the southern group, significant differences were found. Only one meristic, the number of upper bicuspid teeth (BTU), differed between the northern and the southern group: i.e. the groups that include the type localities of *S*. *babaulti* and *S*. *pleurospilus* respectively, whereas all comparisons including the western and the eastern groups differed in at least two of the meristics. Meristic values overlapped between any of the five geographical groups except for the number of scales in the upper lateral line (ULL) (21–22 in the northern vs. 23–24 in the western group), the number of scales in the longitudinal line (LongL) (30.5–31.5 in the northern and the northeastern vs. 32–34 in the western group) and the number of upper bicuspid teeth (BTU) (26–30 in the northern vs. 31–38 in the western group) (not shown). All of the meristics of *S*. *pleurospilus* overlapped with those of *S*. *babaulti*.

**Table 6:** Summary of pairwise Mann-Whithey U tests between 5 groups of *S*. *babaulti* and *S*. *pleurospilus*.

>	N	NE	E	W	S
N	/	PeL*	ULL**, MLL*, LongL***, BTU*, BD**, PeL**	ULL**, LongL**, BTU*	BTU*, LaD*, PeL**
NE	/	/	DSp*, LongL***	DSR*, LongL**, CV*	SnL*
E	/	LGR*	/	DSR**, PrP*, PrV*	LGR*
W	/	BD*	DSp**, PPL*, BD**, DFB**	/	LJL*
S	/	ASL*	DSp**, ULL***, LongL*, AV**	ULL*, LongL*	/

Test were performed on 15 meristics and 20 percentages of measurements (bold) with; N: north, NE: northeast, E: east, W: west and S: south (*S*. *pleurospilus*). Values are significantly larger in groups listed in the columns than in the rows, *, ** and *** denote significance at the 0.05, 0.001 and 0.0001 level after Bonferroni correction.

#### Measurements

**Fig 6:**
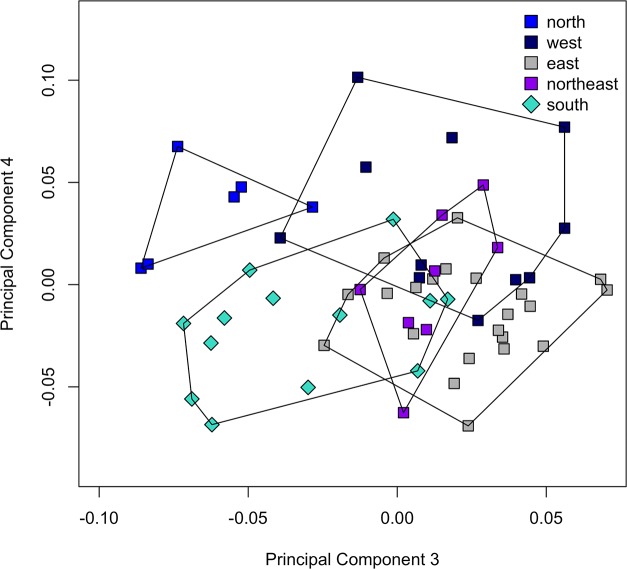
PCA on measurements of *S*. *babaulti* and *S*. *pleurospilus*. PC4 vs. PC3 of a PCA on the 20 measurements belonging to four geographical groups of *S*. *babaulti* and to *S*. *pleurospilus* (the southern group).

A PCA was performed on the covariance matrix of log-transformed measurements taken on *S*. *babaulti* and *S*. *pleurospilus* specimens (Table 7). Here, the second PC did not show any pattern (not shown) and a scatter-plot of the fourth versus the third PC was presented (Fig 6). The third PC incompletely separated the northern *S*. *babaulti* (including the holotype) from all other *S*. *babaulti* groups. Values from the southern group, i.e. *S*. *pleurospilus*, overlapped with all of the other groups. The fourth PC incompletely separated *S*. *pleurospilus* from the northern and western *S*. *babaulti*. Specimens from the northern and western shores had, on average, higher values for this axis than specimens from the eastern and northeastern groups. In spite of what was observed for the meristics, none of the PC allowed for a separation between *S*. *babaulti* specimens from the central eastern and northeastern shores. For PC3, explaining 2.23% of the variance, the mouth (MW) and inter-orbital width (IOW) were the main contributors whereas for PC 4, explaining 1.79%, these were the length of the caudal peduncle (CPL) and of the premaxillary processus (PPL). The differences observed in the exploratory analysis were reflected by pair-wise Mann-Whitney U tests (Table 6). These revealed 14 measurements to differ significantly. Measurements differed significantly between all groups except between the northeastern and the eastern groups. Nevertheless, only one measurement, the length of the pectoral fin, could be used to separate any of the groups (25.39–27.75% in the northern vs. 27.45–34.13%SL in all other groups, with values for only one western specimen overlapping). As for the meristics, all measurements (as %) overlapped between *S*. *pleurospilus* and *S*. *babaulti*.

**Table 7:** PCA on the measurements of *S*. *babaulti* and *S*. *pleurospilus*.

	PC1	PC2	PC3	PC4
VAR (%)	86.4450	4.5187	2.2275	1.7855
SL	0.2044	0.0546	0.0509	0.1799
LaD	0.2853	-0.2767	0.0844	-0.1820
SnL	0.2685	-0.2555	-0.0200	-0.0454
LJL	0.2116	-0.2855	0.0279	-0.3451
PPL	0.2013	0.0571	0.1120	-0.4067
ED	0.1600	-0.1143	0.1756	-0.0152
IOW	0.2846	0.3370	-0.3976	-0.1814
MW	0.3017	-0.1808	-0.6190	0.1903
HL	0.2033	-0.2071	0.0971	-0.0590
BD	0.2289	0.2350	0.0674	-0.3280
PeL	0.2533	0.1329	0.3486	-0.0536
ASL	0.1383	0.1654	0.3422	0.0870
DFB	0.2099	0.3223	0.0250	0.0723
AFB	0.2178	0.3144	-0.1370	0.0705
PrD	0.2053	-0.0332	0.1016	-0.1620
PrP	0.2017	-0.3307	0.0501	0.2804
PrV	0.2088	-0.2270	0.0587	0.2986
PrA	0.2007	-0.0612	0.0643	0.2257
CPL	0.1884	0.2446	0.2814	0.4368
CPD	0.2262	0.1991	-0.1815	0.1049

Loadings and explained variance of the first four PC of a PCA conducted on the covariance matrix of 20 log-transformed measurements taken on 60 specimens.

### Host genetics: barcoding

Inter- and intra-specific distances for *Simochromis*, *Pseudosimochromis* and other “sediment dwelling” tropheine species are compared in Table 8. Sequences of the commonly used barcoding gene COI show a clear distinction between the within-species and the between-species level for all analyzed cichlids. *Simochromis babaulti* displays remarkably higher intra-specific genetic variation. Its difference with *S*. *pleurospilus* is of the same order of magnitude as the genetic distances within *S*. *babaulti*. As mitochondrial markers are not reliable to infer interspecific historical relationships in these fishes [39], these barcoding data are only used to compare genetic distances within and between species, without an attempt at phylogenetic reconstruction.

**Table 8:** Barcoding results for *Pseudosimochromis*, *Simochromis* and the other “sediment dwelling” tropheine genera.

	intraspecific	‘*C*.’ *horei*	‘*G*.’ *pfefferi*	*L*. *dardennii*	*P*. *curvifrons*	*S*. *babaulti*	*S*. *diagramma*	*S*. *marginatus*
**‘*C*.’ *horei***	0.6–1.4							
**‘*G*.’ *pfefferi***	0–1.3	7.1–10.1						
***L*. *dardennii***	0.3	1.9–2.7	5.9–7.1					
***P*. *curvifrons***	0.2	3.4–4.2	7.5–9.2	3.2–3.5				
***S*. *babaulti***	0–3.8	3.5–5.5	7.6–9.8	3.3–4.4	2.9–4.4			
***S*. *diagramma***	0.2–0.8	4.4–5.7	8.5–10.1	3.9–5.2	5.3–6.4	4.3–6.3		
***S*. *marginatus***	0–0.5	4.6–6.9	9.4–11.3	4.7–5.7	6.1–7.0	5.7–7.6	6.3–7.9	
***S*. *pleurospilus***	0	4.9–5.8	9.7–10.2	4.7	4.4–4.7	0.7–4.0	5.8–6.4	7.3–7.9

Comparison of intra- (left column) and inter-specific (minimum-maximum) gamma-corrected pairwise genetic distances (in %) for COI.

### Parasitology

**Fig 7:**
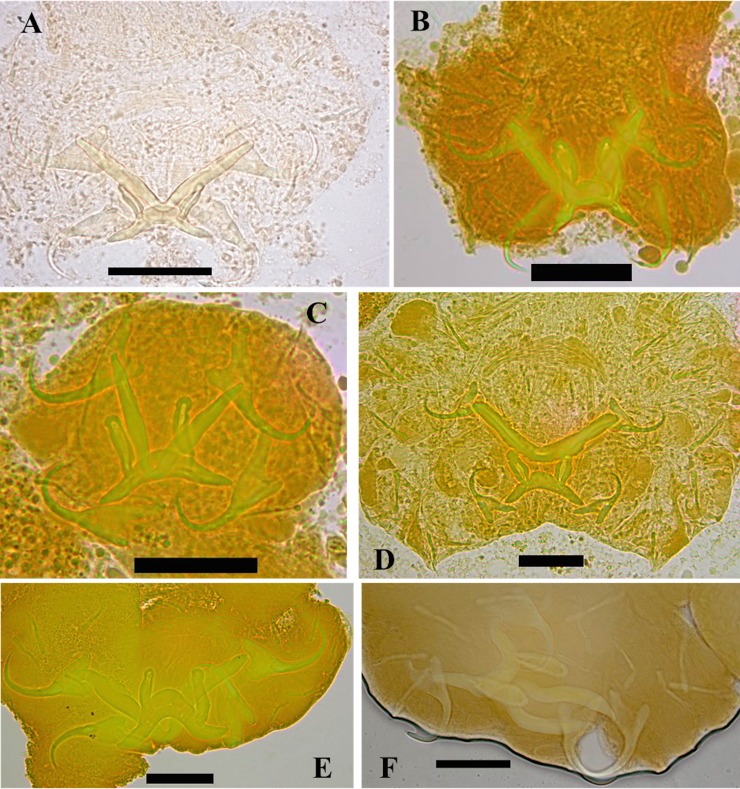
Haptoral morphology of the newly described *Cichlidogyrus* species infecting *Simochromis* and *Pseudosimochromis* cichlids. Micrographs depict the attachment organ of **A.** *C*. *raeymaekersi* **sp. nov. B.** *C*. *muterezii* **sp. nov. C.** *C*. *banyankimbonai* **sp. nov. D.** *C*. *georgesmertensi* **sp. nov. E.** *C*. *franswittei* **sp. nov. F.** *C*. *frankwillemsi* **sp. nov.** Scale bar = 20 μm (A, F) or 30 μm (B, C, D, E).

**Fig 8:**
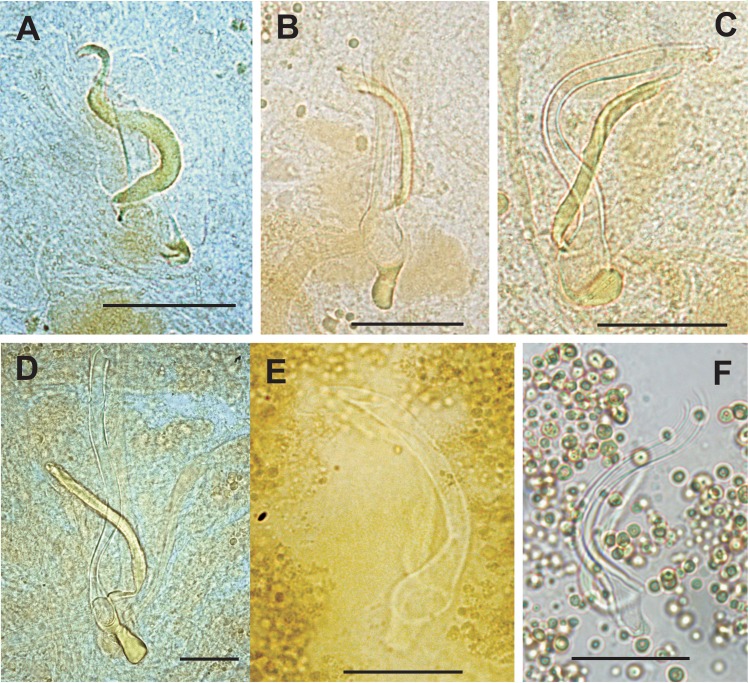
Male genital morphology of the newly described *Cichlidogyrus* species infecting *Simochromis* and *Pseudosimochromis* cichlids. Micrographs depict the male copulatory organ of **A.** *C*. *raeymaekersi* **sp. nov. B.** *C*. *muterezii* **sp. nov. C.** *C*. *banyankimbonai* **sp. nov. D.** *C*. *georgesmertensi* **sp. nov. E.** *C*. *franswittei* **sp. nov. F.** *C*. *frankwillemsi* **sp. nov.** Scale bar = 20 μm.

Monogeneans were collected from the gills of *S*. *diagramma*, *S*. *babaulti*, *S*. *pleurospilus*, *S*. *marginatus* and *P*. *curvifrons*. Investigation of the two *S*. *margaretae* paratypes did not yield any monogenean gill parasites. All monogeneans belong to *Cichlidogyrus* Paperna, 1960 (sensu [83] and [84]): Ancyrocephalidae (but see Introduction), and are new to science; six species are described below (Figs 7 and 8; Table 9). Note that the authors of the new taxa are different from the authors of this paper; Article 50.1 and Recommendation 50A of the International Code of Zoological Nomenclature.

**Table 9:** Measurements of *Cichlidogyrus* species infecting *Simochromis* and *Pseudosimochromis* species.

		*C*. *raeymaekersi* sp. nov.	*C*. *muterezii* sp. nov.	*C*. *banyankimbonai* sp. nov.	*C*. *georgesmertensi* sp. nov.	*C*. *franswittei* sp. nov.	*C*. *frankwillemsi* sp. nov.
AS	L	628 ± 86.8 (430.1–812.9) _**25**_	536 ± 149.4 (354.2–777.4) _**11**_	516 ± 150.6 (304.1–686.1) _**9**_	1111 ± 202.6 (744.3–1520.2) _**25**_	388 ± 47.0 (307.5–509.9) _**20**_	307 ± 66.9 (171.3–406.8) _**13**_
	W	124.4 ± 17.5 (91.4–156.9) _**25**_	122.8 ± 25.5 (84.6–159.9) _**11**_	121.8 ± 36.4 (67.6–164.3) _**10**_	231.8 ± 40.6 (165.0–309.2) _**25**_	84.2 ± 19.4 (55.4–140.4) _**20**_	100.4 ± 33.4 (68.0–182.4) _**13**_
Ph	W	38.4 ± 4.2 (30.8–47.1) _25_	59.3 ± 7.7 (47.3–71.4) _**12**_	42.8 ± 6.3 (32.3–49.3) _**11**_	72.9 ± 13.0 (37.3–92.0) _**25**_	26.5 ± 4.3 (20.8–36.9) _**20**_	28.1 ± 7.8 (20.3–47.1) _**11**_
DA	a	39.1 ± 5.7 (29.9–46.4) _50_	27.5 ± 1.6 (24.2–30.6) _**22**_	30.4 ± 2.5 (25.9–35.1) _**22**_	28.3 ± 2.2 (19.1–32.7) _**49**_	35.1 ± 2.5 (30.8–39.5) _**30**_	22.1 ± 0.7 (20.5–23.4) _**27**_
	b	27.7 ± 3.9 (20.3–33.1) _49_	22.0 ± 1.2 (19.4–24.1) _**22**_	22.7 ± 2.1 (18.8–26.6) _**22**_	20.2 ± 1.2 (17.7–22.9) _**48**_	24.7 ± 1.5 (22.2–28.7) _**30**_	18.8 ± 0.7 (17.4–20.0) _**27**_
	c	3.4 ± 0.9 (1.7–5.1) _49_	3.8 ± 1.1 (2.1–5.8) _**22**_	3.3 ± 0.6 (2.4–4.7) _**22**_	5.2 ± 0.8 (3.6–7.0) _**48**_	4.0 ± 0.7 (2.5–5.2) _**30**_	4.0 ± 0.6 (3.2–5.1) _**27**_
	d	14.3 ± 2.1 (10.2–18.0) _49_	8.9 ± 1.2 (6.7–11.8) _**22**_	11.0 ± 1.3 (9.2–13.9) _**22**_	11.3 ± 1.4 (8.6–15.4) _**49**_	14.2 ± 1.9 (9.3–17.2) _**30**_	7.5 ± 0.6 (6.2–8.8) _**27**_
	e	10.7 ± 1.1 (8.4–13.4) _50_	7.8 ± 0.9 (6.2–10.1) _**22**_	9.0 ± 0.8 (6.4–10.1) _**22**_	7.5 ± 0.7 (6.2–9.5) _**49**_	10.1 ± 1.2 (7.8–12.2) _**30**_	7.7 ± 0.8 (5.9–10.2) _**27**_
DB	x	37.6 ± 5.1 (29.3–46.0) _25_	30.0 ± 3.8 (23.7–36.6) _**12**_	35.3 ± 3.3 (30.1–41.5) _**12**_	36.5 ± 3.3 (30.6–44.4) _**25**_	33.9 ± 4.5 (23.2–40.8) _**20**_	23.9 ± 3.6 (19.7–29.1) _**8**_
	y	12.7 ± 2.6 (9.0–19.0) _25_	11.0 ± 1.5 (9.5–14.8) _**12**_	10.7 ± 1.6 (8.8–13.5) _**12**_	12.5 ± 1.2 (10.4–15.0) _**24**_	12.3 ± 2.4 (7.9–16.9) _**20**_	8.8 ± 1.6 (7.0–11.0) _**10**_
	w	8.8 ± 2.1 (5.0–12.2) _25_	6.4 ± 1.0 (4.1–7.7) _**12**_	7.1 ± 1.1 (5.7–9.3) _**12**_	6.6 ± 0.9 (5.0–8.0) _**25**_	6.4 ± 1.1 (4.1–8.9) _**20**_	4.1 ± 0.9 (3.0–5.4) _**10**_
	h	13.8 ± 2.2 (9.6–18.5) _49_	16.0 ± 2.5 (12.5–22.9) _**24**_	15.5 ± 2.9 (10.5–20.9) _**24**_	15.2 ± 2.1 (8.5–20.1) _**48**_	12.3 ± 1.3 (9.5–15.0) _**33**_	11.8 ± 1.5 (8.8–14.0) _**19**_
VA	a	31.8 ± 3.2 (26.1–36.2) _50_	29.5 ± 1.0 (27.3–31.5) _**20**_	31.2 ± 2.2 (28.0–35.6) _**24**_	27.9 ± 1.4 (24.5–30.5) _**50**_	32.0 ± 1.7 (28.3–35.5) _**31**_	23.7 ± 0.7 (22.0–24.6) _**26**_
	b	30.5 ± 3.3 (24.3–35.4) _49_	25.8 ± 1.0 (23.5–28.3) _**20**_	25.6 ± 1.9 (22.7–28.9) _**24**_	23.9 ± 1.4 (20.8–27.2) _**50**_	28.9 ± 1.4 (26.5–31.5) _**31**_	21.5 ± 0.8 (19.6–23.3) _**26**_
	c	3.7 ± 1.1 (1.8–6.2) _49_	4.0 ± 0.9 (2.4–5.6) _**20**_	3.6 ± 0.8 (2.1–5.0) _**24**_	5.7 ± 0.9 (3.4–7.7) _**50**_	4.3 ± 0.7 (2.8–5.5) _**31**_	3.4 ± 0.5 (2.6–4.2) _**26**_
	d	9.3 ± 1.6 (5.7–13.9) _49_	8.2 ± 1.2 (5.9–10.7) _**20**_	9.9 ± 1.3 (7.7–13.0) _**24**_	9.1 ± 1.1 (6.4–11.5) _**50**_	10.1 ± 1.3 (8.0–12.7) _**31**_	7.5 ± 0.7 (6.1–9.1) _**26**_
	e	11.2 ± 0.9 (9.5–13.3) _50_	9.7 ± 0.9 (7.4–11.0) _**20**_	10.6 ± 0.7 (8.7–11.7) _**24**_	8.7 ± 0.8 (6.7–10.1) _**50**_	12.0 ± 1.2 (9.2–14.4) _**31**_	8.9 ± 1.1 (4.5–10.1) _**26**_
VB	x	38.8 ± 5.0 (30.0–47.2) _50_	32.7 ± 2.7 (27.8–38.0) _**24**_	33.9 ± 3.0 (30.1–40.5) _**24**_	40.4 ± 2.9 (33.6–46.3) _**48**_	33.5 ± 3.0 (28.9–42.4) _**34**_	26.4 ± 2.4 (22.2–29.6) _**17**_
	w	7.5 ± 1.4 (4.9–10.1) _25_	5.7 ± 0.9 (3.8–6.7) _**12**_	5.8 ± 0.9 (4.8–7.4) _**12**_	6.4 ± 0.9 (5.1–8.8) _**25**_	5.6 ± 0.6 (4.5–6.9) _**18**_	3.1 ± 0.6 (2.5–4.0) _**9**_
U	I	11.9 ± 0.4 (11.2–12.9) _50_	12.5 ± 0.6 (10.7–13.2) _**20**_	12.3 ± 0.5 (11.6–13.7) _**23**_	11.9 ± 0.9 (10.1–14.4) _**47**_	11.9 ± 0.6 (11.0–13.6) _**36**_	11.4 ± 0.6 (10.2–12.8) _**19**_
	II	10.7 ± 0.3 (10.1–11.9) _47_	11.7 ± 0.4 (10.8–12.4) _**16**_	11.5 ± 0.3 (11.0–12.1) _**17**_	11.2 ± 0.7 (9.4–12.6) _**44**_	10.6 ± 1.0 (8.9–11.7) _**11**_	10.7 ± 0.4 (10.0–11.3) _**16**_
	III-VII	16.3 ± 2.0 (12.1–20.9) _229_	22.5 ± 1.6 (19.2–27.1) _**101**_	15.9 ± 2.3 (12.1–20.8) _**101**_	21.7 ± 2.1 (15.6–27.2) _**205**_	18.2 ± 2.6 (13.4–25.0) _**95**_	18.1 ± 1.4 (15.2–22.4) _**103**_
MA	He	2.3 ± 0.6 (1.3–4.0) _25_	7.0 ± 1.2 (5.1–8.4) _**12**_	4.1 ± 1.0 (2.1–5.7) _**12**_	11.3 ± 2.6 (7.1–15.8) _**25**_	5.4 ± 1.5 (3.1–9.1) _**20**_	0.9 ± 0.7 (0.0–1.8) _**16**_
	Pe	29.3 ± 1.4 (27.2–32.4) _25_	42.4 ± 4.6 (37.5–50.3) _**12**_	58.7 ± 3.5 (54.1–65.1) _**12**_	92.2 ± 2.6 (88.0–96.9) _**25**_	53.5 ± 2.4 (47.1–57.0) _**20**_	49.2 ± 3.1 (43.7–54.6) _**16**_
	Ap	25.5 ± 1.5 (23.2–30.0) _25_	29.5 ± 3.0 (24.4–33.3) _**12**_	33.5 ± 1.5 (30.7–35.5) _**12**_	60.7 ± 2.7 (53.1–64.8) _**25**_	36.0 ± 3.6 (30.7–46.1) _**20**_	31.3 ± 1.7 (28.2–34.6) _**16**_

Summary of measurments [average ± standard deviation (minimum, maximum) all in μm], with AS: Adult size, L: length, W: width, Ph: Pharynx, DA: dorsal anchor, DB: dorsal bar, VA: ventral anchor, VB: ventral bar, U: uniculi and MA: male apparatus, other abbrevations as in Fig 1, subscripts denote the number of specimens examined.

### Species Descriptions

**Fig 9:**
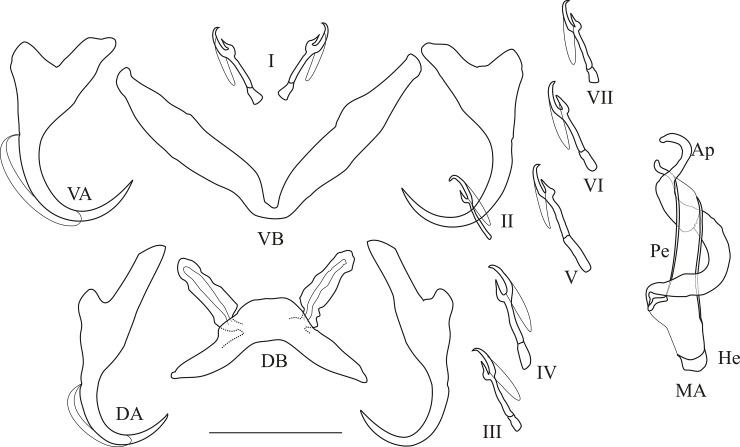
Haptoral and genital hard parts of *Cichlidogyrus raeymaekersi* sp. nov. (Ap) accessory piece (DB) dorsal transverse bar, (DA) dorsal anchor, (He) heel, (MA) male apparatus, (Pe) penis, (VB) = ventral transverse bar, (VA) ventral anchor, (I) to (VII) uncinuli, scale bar = 20 μm.

*Cichlidogyrus raeymaekersi* Pariselle & Vanhove **sp. nov.** (Figs 7A, 8A and 9; Table 9)

urn:lsid:zoobank.org:act:DF18D90F-7103-496C-ADF8-3C23AE5922C2

*Type host*: *Simochromis diagramma* (Günther, 1894)

*Infection site*: Gills.

*Type locality*: Mukamba, D.R.Congo (6° 57’ S 29° 43’ E) (April 16^th^, 2010, on MRAC B0-12-P-659 and -662)

*Other localities*: Kabulu, D.R.Congo (6° 40’ S, 29° 30’ E) (April 21^st^, 2010); Kalambo Lodge, Zambia (8° 37’ S 31° 12’ E) (April 18^th^, 2008, on B2-4-P-58-60 (1), B2-4-P-61-64 (2), August 29^th^— 31^st^, 2011, on B1-23-P-376-410); Kapakwe, D.R.Congo (6° 58’ S 29° 44’ E) (April 17^th^, 2010, on MRAC B0-12-P-674 and -677); Katoto, Zambia (08° 48’ S 31° 01' E) (September 12^th^, 15^th^, 2011, on MRAC B1-23-P-479-496); Kyanza, D.R.Congo (7° 7’ S 29° 59’) (April 19^th^, 2010); Mbita Island, Zambia (08° 45’ S 31° 05’ E) (April 10^th^, 2008, on B2-4-P-114-115; September 9^th^, 15^th^, 2011, on B1-23-P-444-460); Mugayo, D.R.Congo (6° 47’ S 29° 34’ E) (April 11^th^, 2010, on MRAC B0-12-P-357); Muzumwa, Zambia (08° 42’ S 31° 12’ E) (September 3^rd^, 2011, on B1-23-411-443); Tumbi, Zambia (08° 42’ S 30° 55’ E) (August 25^th^, 2011 B1-23-P-324-375); Wonzye Point, Zambia (8° 43’ S 31° 08’ E) (August 23^rd^, 2011, on MRAC B1-23-P-517-549).

*Material studied*: 25 individuals (21 from Mukamba, 2 from Kapakwe and 2 from Mugayo).

*Type material*: holotype: MRAC 37757 (1, Mukamba); paratypes: MRAC 37757 (4, Mukamba); MRAC 37756 (5, Mukamba), MNHN HEL423 (4, Mukamba), SAMCTA 61807 (2, Kapakwe).

*Etymology*: The name *C*. *raeymaekersi* **sp. nov.** is given in honour of Dr. Joost A.M. Raeymaekers (Belgium) for his research on the fish hosts and his help in our work.

*Description*: Haptor: dorsal anchor with short shaft, long guard and arched blade; dorsal transverse bar thick and arched; ventral anchor with marked shaft and guard; ventral transverse bar V-shaped and thick, uncinuli I small (sensu [49], i.e. relative to the length of uncinuli II, the pair which retains its larval length [85]), uncinuli III to VII short (sensu [85]). Penis, beginning in an elongated bulb, with short heel, is a short, straight and wide tube with a bevelled ending. Accessory piece simple and spirally coiled, winds around penis (1.5 turns) and attached by thin filament to distal extremity of basal bulb. No sclerotised vagina observed.

*Comments*: *Cichlidogyrus raeymaekersi* **sp. nov.** belongs to the group with short uncinuli I and III to VII (sensu [49]). This group includes: *C*. *acerbus* Dossou, 1982; *C*. *amieti* Birgi & Euzet, 1983; *C*. *amphoratus* Pariselle & Euzet, 1995; *C*. *berrebii* Pariselle & Euzet, 1994; *C*. *bifurcatus* Paperna, 1960; *C*. *cirratus* Paperna, 1964; *C*. *cubitus* Dossou, 1982; *C*. *fontanai* Pariselle & Euzet, 1997; *C*. *gillardinae* Muterezi Bukinga, Vanhove, Van Steenberge & Pariselle, 2012; *C*. *giostrai* Pariselle, Bilong Bilong & Euzet, 2003; *C*. *gistelincki* Gillardin, Vanhove, Pariselle, Huyse & Volckaert, 2011; *C*. *haplochromii* Paperna & Thurston, 1969; *C*. *irenae* Gillardin, Vanhove, Pariselle, Huyse & Volckaert, 2011; *C*. *karibae* Douëllou, 1993; *C*. *lagoonaris* Paperna, 1969; *C*. *levequei* Pariselle & Euzet, 1996; *C*. *longipenis* Paperna & Thurston, 1969; *C*. *louipaysani* Pariselle & Euzet, 1994; *C*. *makasai* Vanhove, Volckaert & Pariselle, 2011; *C*. *mbirizei* Muterezi Bukinga, Vanhove, Van Steenberge & Pariselle, 2012; *C*. *mulimbwai* Muterezi Bukinga, Vanhove, Van Steenberge & Pariselle, 2012; *C*. *nageus* Řehulková, Mendlová & Šimková, 2013; *C*. *njinei* Pariselle, Bilong Bilong & Euzet, 2003; *C*. *nyongensis* Pariselle, Bitja Nyom & Bilong Bilong, 2013; *C*. *ornatus* Pariselle & Euzet, 1995; *C*. *pouyaudi* Pariselle & Euzet, 1994; *C*. *rognoni* Pariselle, Bilong Bilong & Euzet, 2003; *C*. *sanjeani* Pariselle & Euzet, 1997; *C*. *sclerosus* Paperna & Thurston, 1969; *C*. *slembroucki* Pariselle & Euzet, 1998; *C*. *steenbergei* Gillardin, Vanhove, Pariselle, Huyse & Volckaert, 2011; *C*. *sturmbaueri* Vanhove, Volckaert & Pariselle, 2011; *C*. *tilapiae* Paperna, 1960, *C*. *vandekerkhovei* Vanhove, Volckaert & Pariselle and *C*. *zambezensis* Douëllou, 1993. *Cichlidogyrus raeymaekersi* **sp. nov.** can easily be distinguished from all these species by the shape of its penis and associated accessory piece (short, straight and wide; spirally coiled (1.5 turns), winds around the penis, attached to the basal bulb, respectively). Only *C*. *reversati* Pariselle & Euzet, 2003 resembles C. *raeymaekersi* **sp. nov.** by the S-shaped accessory piece (coiled, winds around the penis, attached), but they can be distinguished by the shape of the ending of the penis (bevelled in *C*. *raeymaekersi* **sp. nov.** versus folded in *C*. *reversati*) and the size of the uncinuli pair I (short in *C*. *raeymaekersi* **sp. nov.** versus large in *C*. *reversati*). *Cichlidogyrus raeymaekersi* **sp. nov.** was previously recorded as *Cichlidogyrus* sp. 1 in [86].

**Fig 10:**
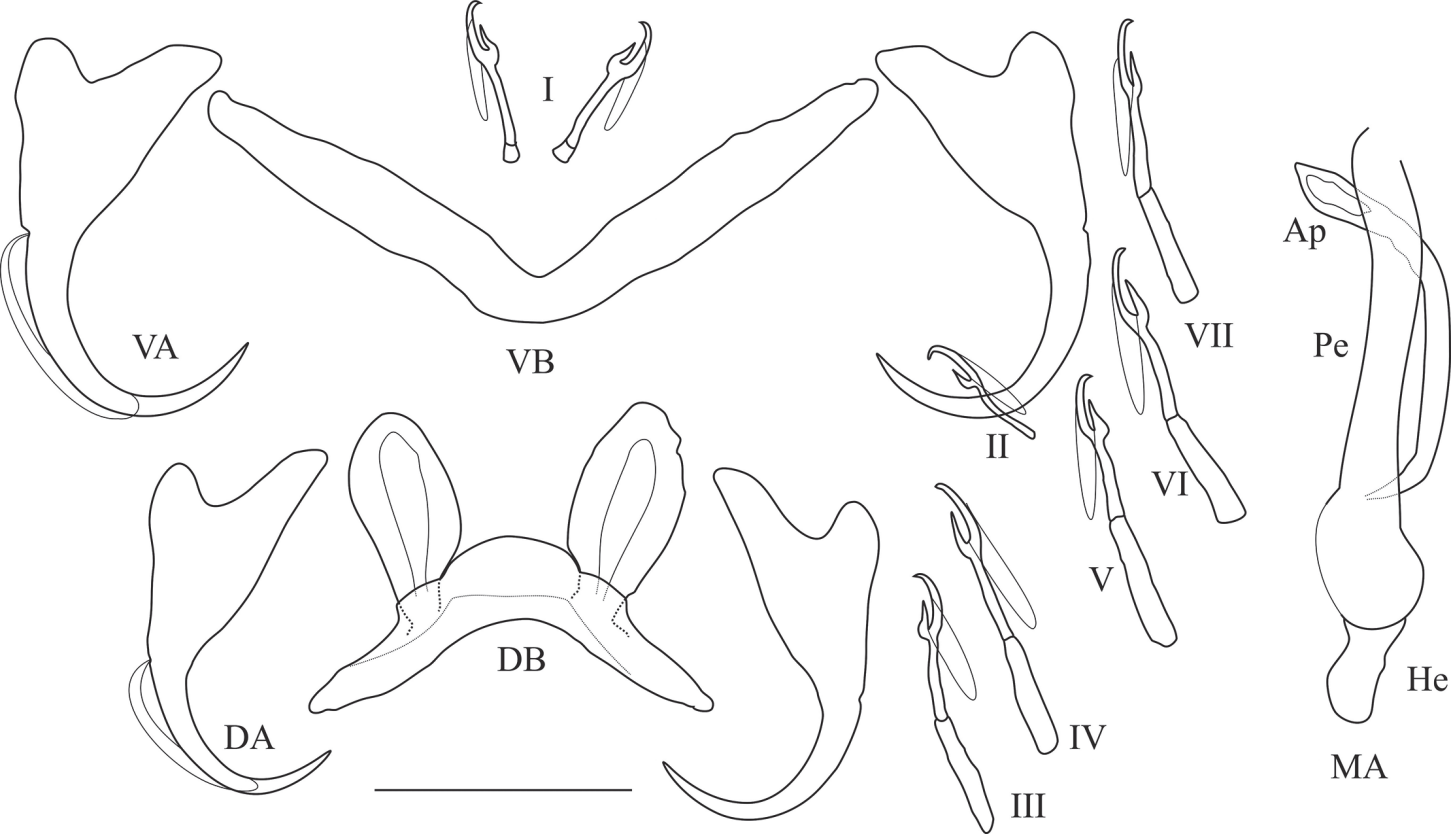
Haptoral and genital hard parts of *Cichlidogyrus muterezii* sp. nov. (Ap) accessory piece (DB) dorsal transverse bar, (DA) dorsal anchor, (He) heel, (MA) male apparatus, (Pe) penis, (VB) = ventral transverse bar, (VA) ventral anchor, (I) to (VII) uncinuli, scale bar = 20 μm.

*Cichlidogyrus muterezii* Pariselle & Vanhove **sp. nov.** (Figs 7B, 8B and 10; Table 9)

urn:lsid:zoobank.org:act:155B5110-BB2E-4314-9F3C-EA47444DAFC9

*Type host*: *Simochromis diagramma* (Günther, 1894).

*Infection site*: Gills.

*Type locality*: Mugayo, D.R.Congo (6° 47’ S 29° 33’ E) (April 11^th^, 2010, on MRAC B0-12-P-357).

*Other localities*: Wonzye Point, Zambia (8° 43’ S 31° 08’ E) (August 23^rd^, 2011, on MRAC B1-23-P-517-549); Mbita Island, Zambia (08° 45’ S 31° 05’ E) (September 9^th^, 15^th^, 2011, on B1-23-P-444-460); Kalemie, D.R.Congo (5° 55’ S 29° 12’ E) (April 23^rd^, 2010, on MRAC B1-12-P-1096); Kisokwe, D.R.Congo (4° 15’ S, 29° 11’ E) (March 23^rd^, 2010, on B0-12-P-966-967 (1)); Mukamba, D.R.Congo (6° 57’ S 29° 43’ E) (April 16^th^, 2010, on B0-12-P-659); Kapakwe, D.R.Congo (6° 53’ S 29° 44’ E) (April 17^th^, 2010, on MRAC B0-12-P-674); Kalambo Lodge, Zambia (8° 37’ S 31° 12’ E) (April 18^th^, 2008, on B2-4-P-58-60 (1), August 30^th^, 2011, on B1-23-P-376-410); Katoto, Zambia (08° 48’ S 31° 01' E) (September 12^th^, 15^th^, 2011, MRAC B1-23-P-479-496); Tumbi, Zambia (08° 42’ S 30° 55’ E) (August 25^th^, 2011 on B1-23-P-342-375); Muzumwa, Zambia (08° 42’ S 31° 12’ E) (September 3^rd^, 2011, on B1-23-411-443).

*Material studied*: 12 individuals (1 from Mugayo, 2 from Mukamba, 2 from Wonzye, 1 from Kalambo, 2 from Katoto and 4 from Muzumwa).

*Type material*: holotype: MRAC 37755 (1, Mugayo); paratypes: MRAC 37754 (1, Katoto); MNHN HEL422 (1, Mukamba).

*Etymology*: The name is given in honour of biologist Fidel Muterezi Bukinga (D.R. Congo), who studies the monogenean fauna of Lake Tanganyika cichlids, for his help in our research.

*Description*: Haptor: dorsal anchor with marked shaft, long guard and arched blade; dorsal transverse bar regularly arched with large auricles; ventral anchor with marked shaft and guard; ventral transverse bar V-shaped; uncinuli I small (sensu [49]), uncinuli III to VII of medium size. Penis, beginning in an oval bulb, with well-developed heel, is a straight and large tube. Accessory piece simple, curved and attached to the basal bulb. No sclerotised vagina observed.

*Comments*: *Cichlidogyrus muterezii* **sp. nov.** belongs to the same group as *C*. *raeymaekersi* **sp. nov.** The former can be distinguished from all the species within this group by the short C-shaped accessory piece and the well-developed heel associated with a straight and large penis. *Cichlidogyrus muterezii* **sp. nov.** was previously recorded as *Cichlidogyrus* sp. 2 in [86].

**Fig 11:**
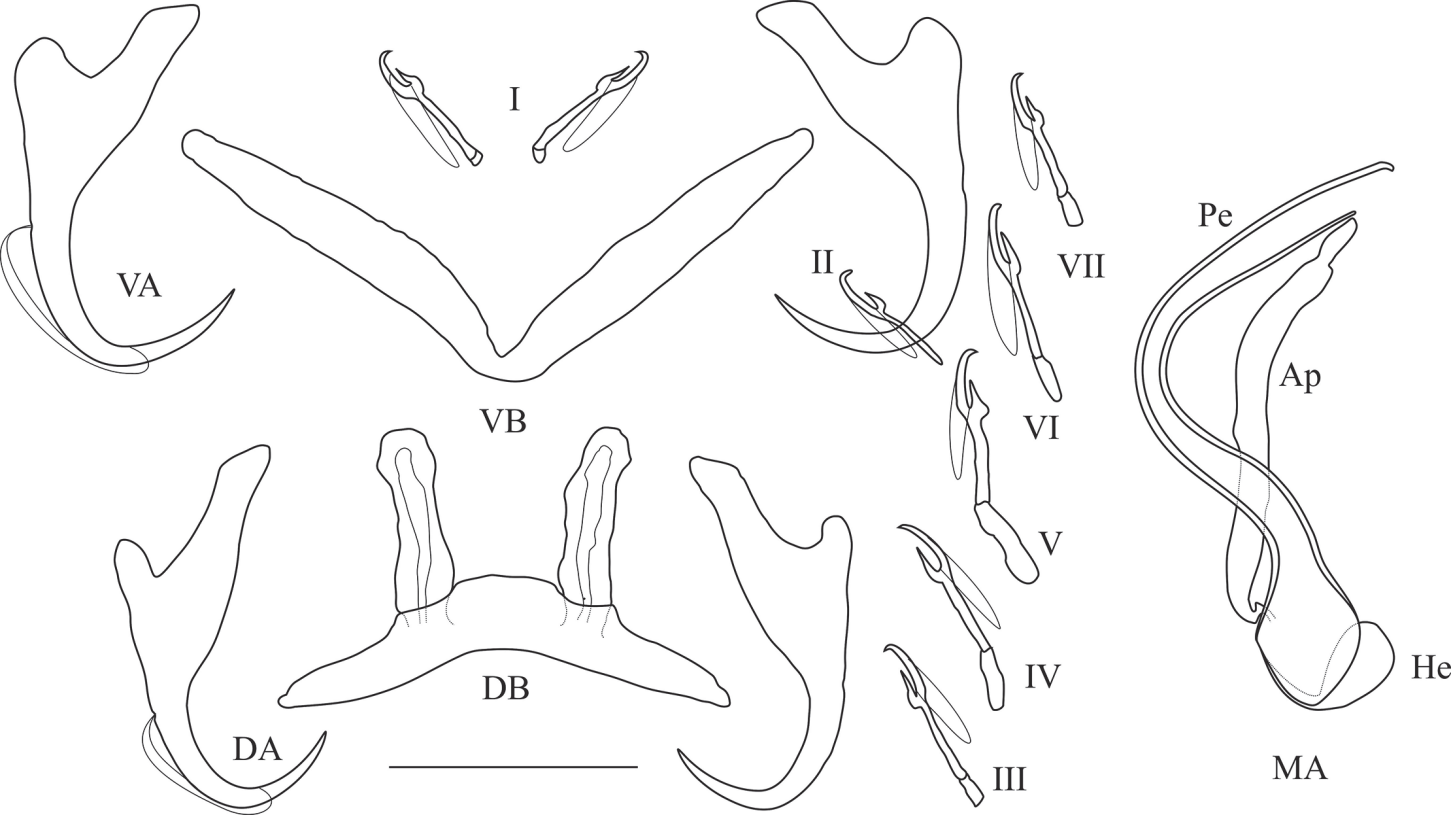
Haptoral and genital hard parts of *Cichlidogyrus banyankimbonai* sp. nov. (Ap) accessory piece (DB) dorsal transverse bar, (DA) dorsal anchor, (He) heel, (MA) male apparatus, (Pe) penis, (VB) = ventral transverse bar, (VA) ventral anchor, (I) to (VII) uncinuli, scale bar = 20 μm.

*Cichlidogyrus banyankimbonai* Pariselle & Vanhove **sp. nov.** (Figs 7C, 8C and 11; Table 9)

urn:lsid:zoobank.org:act:E9C78A9A-7390-4904-81EE-D2B45AEFABEB

*Type host*: *Simochromis diagramma* (Günther, 1894).

*Infection site*: Gills.

*Type locality*: Mukamba, D.R.Congo (6° 57’ S 29° 43’ E) (April 16^th^, 2010, on MRAC B0-12-P-659 and -662).

*Other localities*: Kalemie, D.R.Congo (5° 55’ S 29° 12’ E) (April 23^rd^, 2010, on B0-12-P-1096); Lubumba, D.R.Congo (3° 59’ S, 29° 7’ E) (March 24^th^, 2010, on B0-12-P-621-625 (1)); Luhanga, D.R.Congo (3° 31’ S, 29° 9’ E) (March 27^th^, 2010, on B0-12-P-970); Mukamba, D.R.Congo (6° 57’ S 29° 43’ E) (April 16^th^, 2010, on MRAC B0-12-P-659); Mugayo, D.R.Congo (6° 47’ S 29° 33’ E) (April 11^th^, 2010, on MRAC B0-12-P-356); Kalambo Lodge, Zambia (8° 37’ S 31° 12’ E) (August 29^th^— 31^st^, 2011 on B1-23-P-376-410); Katoto, Zambia (08° 48’ S 31° 01' E) (September 12^th^, 15^th^, 2011, MRAC B1-23-P-479-496); Mbita Island, Zambia (08° 45’ S 31° 05’ E) (September 9^th^, 15^th^, 2011, on B1-23-P-444-460); Muzumwa, Zambia (08° 42’ S 31° 12’ E) (September 3^rd^, 2011, on B1-23-411-443); Tumbi, Zambia (08° 42’ S 30° 55’ E) (August 25^th^, 2011 on B1-23-P-342-375); Wonzye Point, Zambia (8° 43’ S 31° 08’ E) (August 23^rd^, 2011, on MRAC B1-23-P-517-549).

*Material studied*: 12 individuals, (3 from Mukamba, 2 from Katoto, 2 from Mbita, 1 from Wonzye, 2 from Tumbi and 2 from Kalambo Lodge).

*Type material*: holotype: MRAC 37753 (1, Mukamba); paratypes: MRAC 37752 (1, Mukamba); MNHN HEL421 (1, Mukamba).

*Etymology*: The name is given in honour of biologist Dr. Gaspard Caporal Banyankimbona (Burundi), a former office mate of the authors in Tervuren and Leuven, for his contributions to our research and to Burundese and African ichthyology.

*Description*: Haptor: dorsal anchor with marked shaft, long guard and arched blade; dorsal transverse bar slightly arched; ventral anchor with marked shaft and guard; ventral transverse bar V-shaped; uncinuli I small (sensu [85]), uncinuli III to VII short (sensu [49]). Penis, beginning in an oval bulb, the proximal end of which is covered by wide and short heel, is a large C-shaped tube with thick walls and constant diameter. Accessory piece long, simple and slightly curved, shorter than penis and ending in slightly constricted portion. Accessory piece attached by its thin beginning to the basal bulb. No sclerotized vagina observed.

*Comments*: *Cichlidogyrus banyankimbonai* **sp. nov.** belongs to the same group as *C*. *raeymaekersi* **sp. nov.** and *C*. *muterezii* **sp. nov.** It can be distinguished from the other species in this group by the C-shaped and large penis, the short heel covering the basal bulb’s proximal extremity, and the simple accessory piece. It resembles *C*. *muterezii* **sp. nov.**, but can be distinguished by the length of the penis (59 versus 42), the length of uncinuli pairs III to VII (16 versus 23) and the length and shape of the heel (4 versus 7, well-developed versus only covering the bulb’s extremity). The only other *Cichlidogyrus* species in this group that also have a wide and C-shaped penis and a simple accessory piece that is shorter than the penis and narrows down towards the distal extremity are *C*. *gillardinae*, *C*. *irenae* and *C*. *steenbergei*. These species infect other tropheine (*C*. *irenae* and *C*. *steenbergei*) or haplochromine (*C*. *gillardinae*) cichlids of Lake Tanganyika. *Cichlidogyrus banyankimbonai* **sp. nov**. can be distinguished from *C*. *gillardinae* by the length and the thickness of the wall of the penis (59 and thick in *C*. *banyankimbonai* **sp. nov.** versus 47 and thin in *C*. *gillardinae*), from *C*. *irenae* by the length and the diameter of the penis (59 and wide in *C*. *banyankimbonai* **sp. nov.** versus 69.5 and very large with a swollen portion in *C*. *irenae*) and from *C*. *steenbergei* by the length, the thickness of the wall and the diameter of the penis and the surface of its basal bulb (59, thick, wide and smooth in *C*. *banyankimbonai* **sp. nov.** versus 63, thin, very large and striated in *C*. *steenbergei*).

*Cichlidogyrus banyankimbonai* **sp. nov.** was previously recorded as *Cichlidogyrus* sp. 3 in [86].

**Fig 12:**
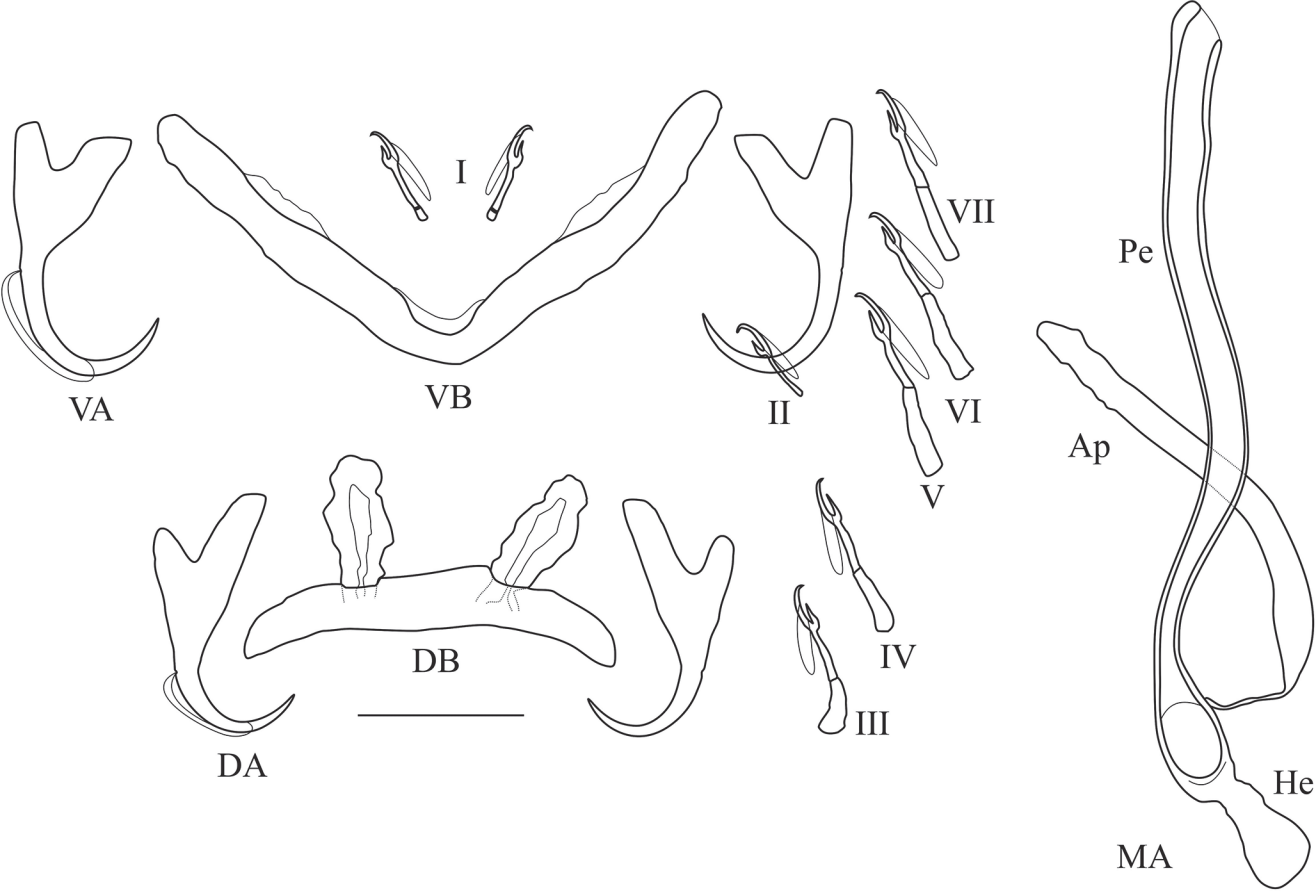
Haptoral and genital hard parts of *Cichlidogyrus georgesmertensi* sp. nov. (Ap) accessory piece (DB) dorsal transverse bar, (DA) dorsal anchor, (He) heel, (MA) male apparatus, (Pe) penis, (VB) = ventral transverse bar, (VA) ventral anchor, (I) to (VII) uncinuli, scale bar = 20 μm.

*Cichlidogyrus georgesmertensi* Pariselle & Vanhove **sp. nov.** (Figs 7D, 8D and 12; Table 9)

urn:lsid:zoobank.org:act:A8C8782C-F8F5-4003-A076-460B0897B3B3

*Type host*: *Simochromis babaulti* (Pellegrin, 1927).

*Other host*: *Simochromis pleurospilus* Nelissen, 1978.

*Infection site*: Gills.

*Type locality*: Kyanza, D.R.Congo (7° 07’ S 29° 59’ E) (April 19^th^, 2010, on MRAC B0-12-P-409, *S*. *babaulti*)

*Other localities*: On *S*. *babaulti*: Bemba, D.R.Congo (3° 37’ S 29° 09’ E) (March 26^th^, 2010, on B0-12-P-846); Lukuga outflow, Kalemie, D.R.Congo (5° 55’ S 29° 11’ E) (April 24^th^, 2010, on B0-12-P-1089); Kabulu, D.R.Congo (6° 40’ S, 29° 30’ E) (April 21^st^, 2010, on B0-12-P-849-850); Kikoti, D.R.Congo (7° 11’ S 30° 04’ E) (April 20^th^, 2010, on B0-12-P-427); Mufazi, D.R.Congo (7° 05’ S 29° 55’ E) (April 13^th^, 2010, on B0-12-P-816-829 (1)). On *S*. *pleurospilus*: Kalambo Lodge, Zambia (8°37’ S 31° 12’ E) (April 16^th^, 2008 on B2-04-P-70-79 (1)); Kama Bay, Zambia (8° 30’ S 30° 40’ E) (January 6^th^, 1976, on MRAC 76-4-P-464-465, paratypes of *S*. *pleurospilus*); Wonzye Point, Zambia (08° 44’ S 31° 08’ E) (April 12^th^, 2008 on B2-04-P-69).

*Material studied*: 25 individuals, all from type locality.

*Type material*: holotype: MRAC 37751 (1, Kyanza); paratypes: MRAC 37751 (4, Kyanza), MRAC 37750 (5, Kyanza), MNHN HEL420 (5, Kyanza), SAMCTA 61808 (5, Kyanza).

*Etymology*: The species epithet, *georgesmertensi*, honours linguist Georges Mertens (Belgium), former Kiswahili lecturer to M.P.M.V. and M.V.S., for his contributions to spreading the knowledge of this language.

*Description*: Haptor: dorsal anchor with marked shaft, long guard and arched blade; dorsal transverse bar slightly arched; ventral anchor with marked shaft and guard; ventral transverse bar V-shaped; uncinuli I small (sensu [85]), uncinuli III to VII of medium size (sensu [85]). Penis, beginning in an oval bulb, with well-developed and club-shaped heel is a long and slightly sinuous tube, of which diameter is larger at the extremity than at the beginning and with thick walls. Accessory piece simple and slightly curved and attached by a filament to basal bulb. No sclerotized vagina observed.

*Comments*: *Cichlidogyrus georgesmertensi* **sp. nov.** belongs to the same group as *C*. *raeymaekersi* **sp. nov.**, *C*. *muterezii* **sp. nov.** and *C*. *banyankimbonai* **sp. nov.** It can be distinguished from all the species in this group by the length and diameter of the penis (long and thick walled) and that of the heel (club-shaped and long). The host specimen investigated from Kama Bay also harboured other monogenean gill parasites not identified to species level (belonging to *Gyrodactylus* and *Cichlidogyrus*). Its *C*. *georgesmertensi* **sp. nov.** displayed a MA and haptoral connective bars that are larger than in the type series. Both anomalies are not further considered here since the host individual, which was collected by Brichard [66] could have been kept in captivity together with other fish species, a condition that could have influenced monogenean development and host range (e.g. [87]).

**Fig 13:**
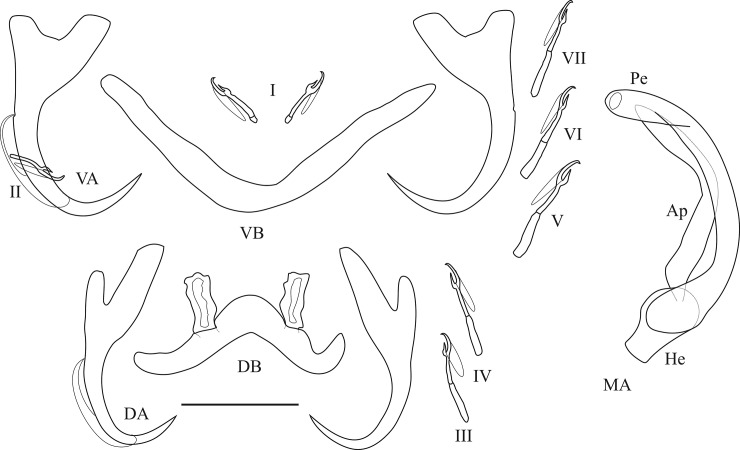
Haptoral and genital hard parts of *Cichlidogyrus franswittei* sp. nov. (Ap) accessory piece (DB) dorsal transverse bar, (DA) dorsal anchor, (He) heel, (MA) male apparatus, (Pe) penis, (VB) = ventral transverse bar, (VA) ventral anchor, (I) to (VII) uncinuli, scale bar = 20 µm.

*Cichlidogyrus franswittei* Pariselle and Vanhove **sp. nov.** (Figs 7E, 8F and 13; Table 9)

urn:lsid:zoobank.org:act:293F7CC7-70B8-469A-B2AA-F22AB1E02131

*Type host*: *Simochromis marginatus* (Poll, 1956).

*Other host*: *Pseudosimochromis curvifrons* (Poll, 1942).

*Infection site*: Gills.

*Type locality*: Luhanga, D.R.Congo (3° 31’ S 29° 9’ E) (October 30th, 1957, on MRAC 129678 and 129680), on type host and (March 27^th^, 2010, on B0-12-P-748) on *P*. *curvifrons*.

*Other localities*: On *S*. *marginatus*: Bemba, D.R.Congo (3° 37’ S 29° 09’ E) (March 26^th^, 2010, on B0-12-P-429). On *P*. *curvifrons*: Bemba, D.R.Congo (3° 37’ S 29° 09’ E) (March 26^th^, 2010, on B0-12-P-430); Cape Tembwe, D.R. Congo (6° 30’ S, 29° 25’ E) (April 10^th^, 2010, on B0-12-P-722); Kalambo Lodge, Zambia (8°37’ S 31° 12’ E) (April 18^th^, 2008, on B2-04-P-98-110(1)); Kasakalawe/Chanzimu, Zambia (8° 47’ S 31° 5’ E) (April 13^th^, 2008, on B2-04-P-97); Mugayo North, D.R.Congo (6° 47’ S 29° 34’ E) (April 11^th^, 2010, on B0-12-P-750); Musamba, Tanzania (7° 50’S 30° 47’E) (April 25^th^, 2008).

*Material studied*: 20 individuals, all from type locality.

*Type material*: holotype: MRAC 37749 (1, Luhanga); paratypes: MRAC 37748 (1, Luhanga), MNHN HEL419 (1, Luhanga), SAMCTA 61812 (1, Luhanga).

*Etymology*: the name *C*. *franswittei* **sp. nov.** is given in honour of the late Dr. Frans Witte (1950–2013), biologist (The Netherlands), for his enormous contributions to (Lake Victoria) cichlid research and the kindness and enthusiasm in his teaching and research.

*Description*: Thick and striated tegument. Haptor: dorsal anchor with marked shaft, long guard and arched blade; dorsal transverse bar thick and arched, sometimes W-shaped; ventral anchor with marked guard and shaft; ventral transverse bar V-shaped; uncinuli I small (sensu [85]), uncinuli III to VII short (sensu [85]). Penis, beginning in a marked bulb, with developed heel is a long, wide and curved tube with a thin wall (often creased) and a sub-terminal aperture. Accessory piece simple and C-shaped and attached to middle of basal bulb. No sclerotized vagina observed.

*Comments*: *C*. *franswittei* **sp. nov.** belongs to the same group as *C*. *raeymaekersi* **sp. nov.,** *C*. *muterezii* **sp. nov.**, *C*. *banyankimbonai* **sp. nov.** and *C*. *georgesmertensi* **sp. nov.** It is the only *Cichlidogyrus* species in this group with a long and large penis that has a sub-terminal opening.

**Fig 14:**
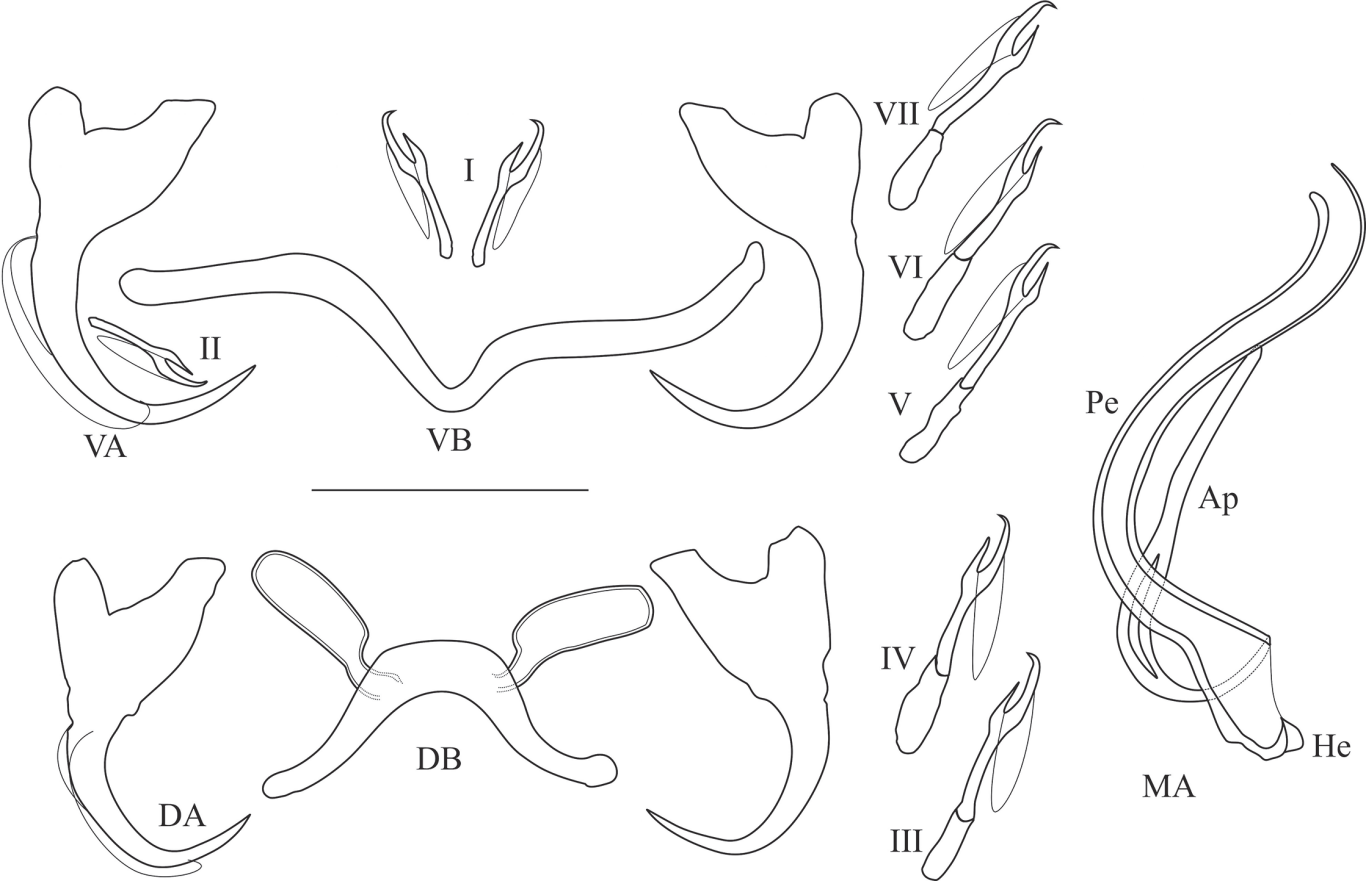
Haptoral and genital hard parts of *Cichlidogyrus frankwillemsi* sp. nov. (Ap) accessory piece (DB) dorsal transverse bar, (DA) dorsal anchor, (He) heel, (MA) male apparatus, (Pe) penis, (VB) = ventral transverse bar, (VA) ventral anchor, (I) to (VII) uncinuli, scale bar = 20 µm.

*Cichlidogyrus frankwillemsi* Pariselle and Vanhove **sp. nov.** (Figs 7F, 8E and 14; Table 9)

urn:lsid:zoobank.org:act:864DD4A0-58A0-4255-BDD1-34316D2D83D5

*Type host*: *Pseudosimochromis curvifrons* (Poll, 1942).

*Infection site*: Gills.

*Type locality*: Kalambo Lodge, Zambia (08°37’ S 31° 12’ E) (April 18^th^, 2008, on B2-04-P-98-110).

*Other localities*: Cape Tembwe, D.R. Congo (6° 30’ S, 29° 25’ E) (April 10^th^, 2010, on B0-12-P-722); Luhanga, D.R.Congo (3° 31’ S 29° 09’ E) (March 27^th^, 2010, on B0-12-P-748)

*Material studied*: 19 individuals, 16 from Kalambo lodge and three from Cape Tembwe.

*Type material*: holotype: MRAC 37747 (1, Kalambo Lodge); paratypes: MRAC 37747 (1, Kalambo Lodge), MRAC 37746 (2, Kalambo Lodge).

*Etymology*: the species epithet, *frankwillemsi*, honours biologist Frank Willems (Zambia / The Netherlands), of the Kasanka Trust for his contributions to community-based conservation and nature education in Africa, in gratitude for his help in our research and travels.

*Description*: Tegument thick and striated. Ovoid pharynx. Haptor: dorsal anchor small with marked shaft and guard and arched blade; dorsal transverse bar thin and slightly sclerotised (often hard to see), arched with thin and well-developed auricles; ventral anchor with marked guard and shaft, slightly larger than dorsal anchor; ventral transverse bar V-shaped, thin and slightly sclerotised (often hard to see); uncinuli I small (sensu [85]), uniculi III to VII short (sensu [85]). Penis, beginning in a slightly marked bulb, with reduced or without heel, is a S-shaped tube with almost constant diameter, its wall thickened at the middle. Accessory piece simple, flattened in the middle, attached to basal bulb and crossing penis proximally. No sclerotized vagina observed.

*Comments*: *Cichlidogyrus frankwillemsi* **sp. nov.** belongs to the same group as *C*. *raeymaekersi* **sp. nov.**, *C*. *muterezii* **sp. nov.**, *C*. *banyankimbonai* **sp. nov.**, *C*. *georgesmertensi* **sp. nov.** and *C*. *franswittei* **sp. nov.** It can be distinguished from all other species in this group, except for *C*. *gistelincki*, by the shape of the penis: S-shaped and thick walled. It differs from *C*. *gistelincki* in the length of the penis (49 in *C*. *frankwillemsi* **sp. nov.** versus 35 in *C*. *gistelincki*) and the shape of the accessory piece (simple and flattened in the middle in *C*. *frankwillemsi* **sp. nov.** versus slender, twisted and with distal end covered by a pointed cap in *C*. *gistelincki*).

## Discussion

We followed an integrative approach to revise the taxonomy of the species currently assigned to *Simochromis* and *Pseudosimochromis*, using both intrinsic (fish morphology and genetics) and extrinsic traits (distribution patterns of monogenean parasitic flatworms). The results presented above confirm the distinction of *S*. *diagramma* from *P*. *curvifrons* and from the ‘small’ *Simochromis* species (*S*. *babaulti*, *S*. *margaretae*, *S*. *marginatus* and *S*. *pleurospilus*). Within the latter group, the specific status of *S*. *pleurospilus* versus that of *S*. *babaulti* is not confirmed (see below) whereas *S*. *margaretae* and *S*. *marginatus* were shown to well-defined species, even though only two specimens of the former species were available for study.

Many quantitative as well as qualitative morphological traits separate *P*. *curvifrons* from *S*. *babaulti*, *S*. *pleurospilus* and *S*. *marginatus*. Yet, for most of these features, *S*. *margaretae* is intermediate between these species and *P*. *curvifrons*. This is the case for the shape of the gill rakers and of the oral teeth, for the distance between the outer and inner teeth and for some meristics, such as the number of anal soft rays. The two *S*. *margaretae* specimens also had values intermediate between those of the other ‘small’ *Simochromis* species and *P*. *curvifrons* in the PCAs of meristics and measurements (Figs 2 and 3). Moreover, *S*. *margaretae* had a steep, highly convex and narrow head as typical for *P*. *curvifrons*. Although some of the traits in which *S*. *diagramma* differs from the ‘small’ *Simochromis* species are also shared by *P*. *curvifrons*, the most clear-cut of them; the absence of reduced gill rakers, can be interpreted as a symplesiomorphism. Indeed, these structures are also absent in other “sediment dwelling” tropheines. Furthermore, *S*. *diagramma* differs clearly in dental morphology from *P*. *curvifrons* and from the ‘small’ *Simochromis* species.

To conclude, qualitative morphological data suggest a closer affinity between *P*. *curvifrons* and the ‘small’ *Simochromis* species than between the ‘small’ *Simochromis* species and *S*. *diagramma*. This is in agreement with a nuclear phylogeny of the Tropheini [39] in which the ‘small’ *Simochromis* species and *P*. *curvifrons* from a monophylum. Unfortunately, *S*. *margaretae* has only been collected once, and no tissue samples, suitable for molecular analysis, are available. Therefore, the intermediate position of this species between *S*. *curvifrons* and the other ‘small’ *Simochromis* species has not been assessed genetically.

The distribution of their *Cichlidogyrus* parasite fauna provided additional evidence for the interrelationships between these cichlids. The haptoral morphology, which was shown to be systematically informative on the scale of *Cichlidogyrus* lineages, is important for the classification of *Cichlidogyrus* species. Details of the genital structures, on the other hand, are mostly useful in distinguishing between closely related *Cichlidogyrus* species [88–89]. Several haptoral features (pronounced asymmetry between shaft and guard in the dorsal anchor, only small uncinuli), as well as some more general characteristics of the genitals (relatively simple accessory piece and copulatory tube, absence of sclerotized vagina) clearly suggest an affinity between the *Cichlidogyrus* species described above with species described from other tropheine cichlids [55,57].

**Fig 15:**
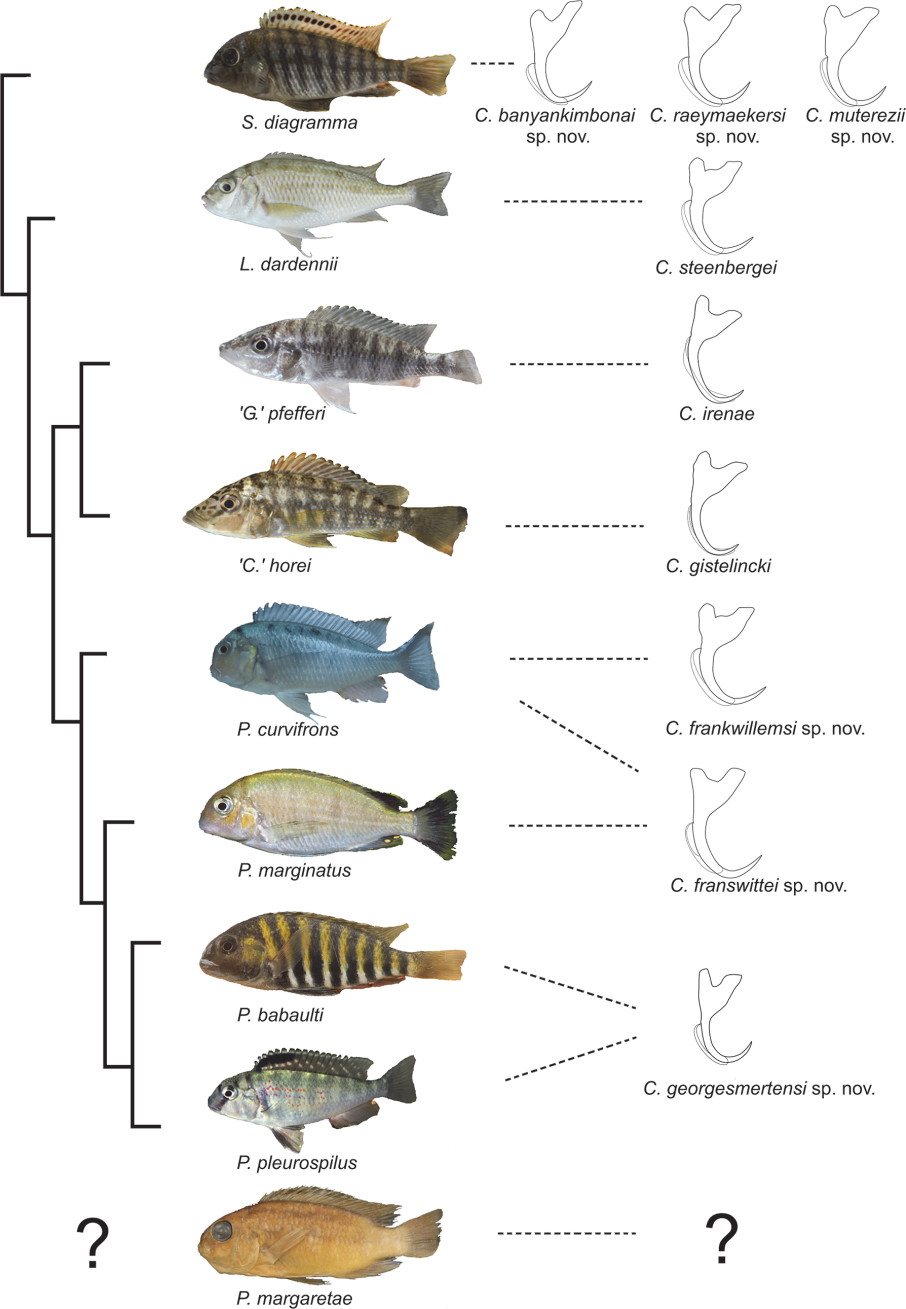
Distribution of *Cichlidogyrus* gill parasites on their tropheine hosts. Phylogenetic reconstruction of the “sediment dwellers” clade within the Tropheini and *S*. *diagramma* is derived from [39]. *Cichlidogyrus* species are visualised by their haptoral ventral anchors, which is a systematically informative trait. Six *Cichlidogyrus* species are described in this paper, three others in [55]. For *P*. *margaretae*, no tissue samples suitable for genetic analysis exist, neither were gill parasites recovered from the available specimens.

When looking at the haptoral morphology of the *Cichlidogyrus* species infecting *Simochromis* and *Pseudosimochromis*, species infecting *S*. *diagramma* were shown to belong to a distinct morphological group. Indeed, *C*. *raeymaekersi* **sp. nov.**, *C*. *muterezii* **sp. nov.** and *C*. *banyankimbonai* **sp. nov.**, found on *S*. *diagramma* **,** all displayed substantial length differences between their ventral anchor guards and shafts. Their dorsal transverse bars were either quite thick or had comparatively large auricles. This clearly sets these three species apart from the species infecting *P*. *curvifrons* and the ‘small’ *Simochromis* species: *C*. *georgesmertensi* **sp. nov.**, *C*. *franswittei* **sp. nov.** and *C*. *frankwillemsi* **sp. nov.** These species had less asymmetrical ventral anchor roots, and either thinner dorsal bars or dorsal bars with relatively small auricles. Likewise, the species infecting the other “sediment-dwelling” tropheines ‘*C*.’ *horei*, ‘*G*.’ *pfefferi* and *L*. *dardennii* also displayed less asymmetry between ventral anchor shaft and guard than the parasites of *S*. *diagramma*. This is in concurrence with *S*. *diagramma* stemming from an early offshoot of this clade. Given the high host specificity in *Cichlidogyrus* infecting tropheine cichlids [45], sharing a *Cichlidogyrus* species may signal a shared ancestry of the cichlid hosts. *Cichlidogyrus franswittei* **sp. nov.** is shared between *P*. *curvifrons* and *S*. *marginatus* whereas *Simochromis babaulti* and *S*. *pleurospilus* share *C*. *georgesmertensi* **sp. nov.** *Cichlidogyrus frankwillemsi* **sp. nov.**, finally, has only been recorded from *P*. *curvifrons* (Fig 15).

The distribution of their *Cichlidogyrus* parasite fauna, combined with the differences in haptoral morphology between the parasites of *S*. *diagramma* and its congeners, provided an additional line of evidence, next to the nuclear phylogeny [39] and the morphology of these cichlids, to justify the generic placement of *P*. *curvifrons* as well as the transfer of all ‘small’ *Simochromis* species: *S*. *babaulti*, *S*. *pleurospilus*, *S*. *marginatus* and *S*. *margaretae* to *Pseudosimochromis*. This renders *Simochromis*, only containing *S*. *diagramma*, monotypic.

The morphological results and barcoding data presented above, however, did not support the specific status of *P*. *pleurospilus* with regard to *P*. *babaulti*. Both species also hosted the same *Cichlidogyrus* species: *C*. *georgesmertensi* **sp. nov.** This also suggested that they are conspecific or at the very least closely related. It is therefore important to consider whether there are genetic or morphological differences between *P*. *pleurospilus* and *P*. *babaulti* warranting their status as two separate species. Although specimens belonging to *P*. *pleurospilus* differed significantly in morphology from geographically separated groups of *P*. *babaulti* populations, these differences were not larger than those observed between the different *P*. *babaulti* groups. This was corroborated by our barcoding results (Table 8). Therefore, *P*. *babaulti* should be considered a Lake Tanganyika species harbouring a considerable amount of geographical variation, with *P*. *pleurospilus* as a junior synonym. A large amount of geographic variation was also observed in different populations of *Neolamprologus niger* (Poll, 1956) [90] and of *Tropheus duboisi* [74]. Finally, this synonymy is in agreement with recent molecular findings. Notwithstanding that a deep split was found in the mitochrondrial phylogeny between *P*. *babaulti* and *P*. *pleurospilus*, AFLP data did not support the monophyly of both species [39].

The reason *P*. *pleurospilus* was originally considered a separate species was that Brichard mentioned the presence of two sympatric but differently coloured *S*. *babaulti*-like species occurring in sympatry at the southern end of Lake Tanganyika [66]. Nevertheless, although Nelissen [66] compared the 13 *S*. *pleurospilus* type specimens with 72 *S*. *babaulti* specimens, none of these *S*. *babaulti* specimens was collected at the same locality as the *S*. *pleurospilus* types. Moreover, according to Brichard’s communications [66], *P*. *babaulti* occurs in the crevices between the rocks whereas *P*. *pleurospilus* can be found in the open (i.e. deeper) water. Taborsky [38] observed that *P*. *babaulti* specimens move to deeper water when they become sexually active, whereas juveniles are found closer to shore. Given that, for *P*. *babaulti*, the colouration pattern changes depending on the sexual or aggressive behaviour of the individual [37], the difference in colour pattern observed by Brichard could be attributed to differences in development or activity and not to the presence of two species. Moreover, in a recent survey of the western lakeshore, populations of mixed colour pattern, encompassing specimens with a colouration typical either to *P*. *babaulti* or to *P*. *pleurospilus*, were found [68].

*Pseudosimochromis*, containing *P*. *curvifrons*, *P*. *babaulti*, *P*. *marginatus* and *P*. *margaretae* can be distinguished from all other tropheine genera by the unique combination of the following characters: three anal spines (versus 4–7 in *Tropheus*), bicuspid firmly set outer anterior oral teeth (versus tricuspid loosly set teeth in *Petrochromis* Boulenger, 1898, bicuspid loosly set teeth in *Interochromis* Yamaoka, Hori & Kuwamura, 1988 and conical or rounded teeth in adult ‘*Ctenochromis*’ *horei*, ‘*Gnathochromis*’ *pfefferi* and *Lobochilotes* Boulenger, 1915) which are densely set (versus gaps present between adjacent anterior oral teeth in *Limnotilapia* Regan, 1920 and in *Simochromis*).

As the largest of the three *S*. *diagramma* syntypes (BMNH 1889–30.9–11) corresponded in size (70. 6mm SL; 90.7mm TL) and in meristics to the specimen illustrated by Günther [91], this specimen is hereby designated as the lectotype, following recommendation ICZN 1999: 74.4. Finally, the synonymy of *Tilapia adolfi* Steindachner, 1909 with *S*. *diagramma* [92] was verified by inspection of six syntypes (NMW 24777–81, of which lot 24777 contains two specimens). Steindachner [93], however, mentioned seven specimens in the species’ original description, whereas only six could be located in the Vienna Museum. The identification of these specimens as *S*. *diagramma* was confirmed and the largest of the six specimens examined: NMW 24777–1 (79.3 mm SL) is hereby designated as the lectotype of *Tilapia adolfi* Steindachner, 1909. It should be noted, however, that *Tilapia adolfi* Steindachner, 1916, is a different species, with replacement name *Tilapia hornorum* Trewavas, 1966, currently a subspecies of *Oreochromis urolepis* (Norman, 1922) [94].

Lake Tanganyika cichlid tribes can be defined on anatomical characters [95] and are supported by molecular studies [22,26]. Within Tropheini, genera are mostly defined by oral morphology and teeth shape, characters linked to feeding habits and ecology. In *Tropheus* and *Pseudosimochromis*, the single row of closely set outer bicuspid teeth (with *Tropheus* having a much broader jaw than *Pseudosimochromis*) reflects their specialisation as browsers. *Simochromis* and *Limnotilapia*, in which large spaces are present between the bicuspid teeth, have a broader ecological niche [59]. The transfer of *S*. *babaulti*, *S*. *marginatus* and *S*. *margaretae* to *Pseudosimochromis* and the fact that its sister clade, consisting of ‘*Gnathochromis*’ *pfefferi* and ‘*Ctenochromis*’ *horei* [39] should change generic position renders six tropheine genera monotypic: *Interochromis*, *Simochromis*, *Limnotilapia*, *Lobochilotes* and ‘*Gnathochromis*’ *pfefferi* and ‘*Ctenochromis*’ *horei*. Remarkably, three of these genera: *Simochromis*, *Lobochilotes* and ‘*Ctenochromis*’ were shown to have a hybrid origin [39]. As, in tropheine cichlids, generic definitions are strongly linked to ecological niche; this suggests that the evolutionary potential to occupy a certain niche was aided by hybridisation. The emergence of evolutionary novelties through hybridisation was suggested by Seehausen [96] and already shown for cichlids from Camerounian Crater Lakes [97]. It could have played a role in the success of Tropheini, as it was suggested that the ancestors of this tribe colonised Lake Tanganyika when other endemic cichlid lineages had already diversified ([32] but see [98]).

The three non-monotypic genera: *Tropheus*, *Pseudosimochromis* and *Petrochromis* all show a considerable amount of morphological variation; the former two were shown to be monophyletic using nuclear markers. Using mitochondrial markers, monophyly could not be obtained for *Tropheus* and *Pseudosimochromis* as the inclusion of *T*. *duboisi* Marlier, 1958 and *P*. *curvifrons* in their genus was not supported [39]. Also from a morphological and an ecological point of view, these two species represent the odd one out within their genus. *Tropheus duboisi* is considered a primitive *Tropheus* species, with a less specialised oral morphology. Previously, it was suggested that *P*. *curvifrons* was a highly specialised algal browser, restricted to the rocky shore [69], whereas its congeners have a more general cichlid bauplan [99] and are less stenotypic. Muschick et al. [26], however, found vegetal (aufwuchs) as well as animal (insects and crustaceans) remains in the guts of *P*. *curvifrons*, which contradicts a specialised life style. Moreover, the species’ stable isotope profile [26] also showed *P*. *curvifrons* to be a generalist. For *P*. *babaulti*, however, guts contained almost 100% vegetal material, showing a specialised ecology. In contrast to *Tropheus*, where there is no intermediate species between the ‘primitive’ *T*. *duboisi* and the specialised other species, an intermediate phenotype exists between that of *P*. *curvifrons* and that of *P*. *babaulti* and *P*. *marginatus*: *P*. *margaretae*. This species was collected in a well-vegetated area [69], which deviates from the rocky shore at which *P*. *curvifrons* is found and which corresponds more with the preferred diet of *P*. *babaulti*.

**Fig 16:**
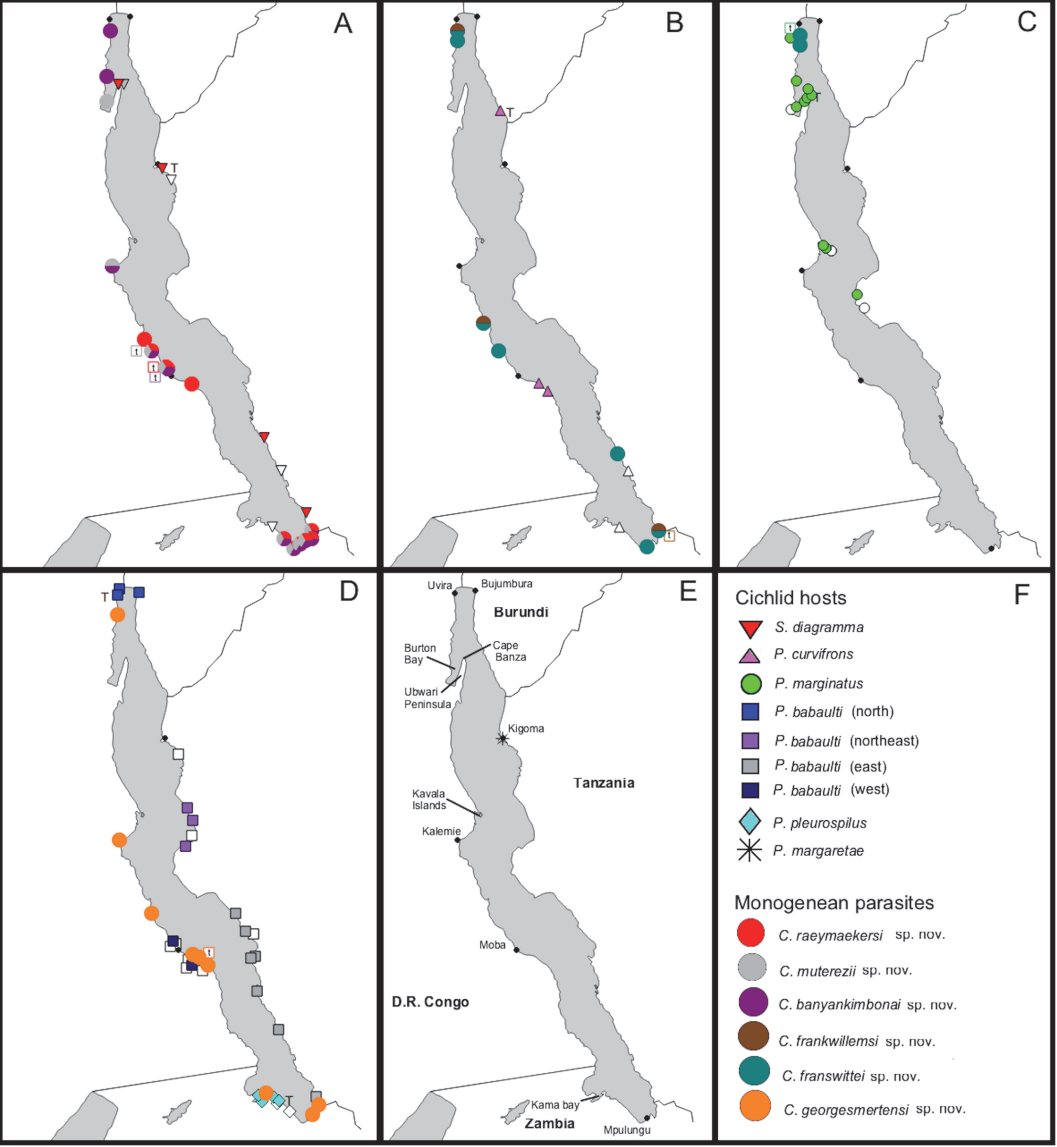
Map of Lake Tanganyika with an overview of specimens examined. Collection localities of hosts and parasites with: filled symbols: cichlids used for morphology, empty symbols: cichlids used for barcoding, pie charts: collection sites of *Cichlidogyrus* parasites, T: cichlid type locality and t: *Cichlidogyrus* type locality, for **A.** *Simochromis diagramma*, **B.** *Pseudosimochromis curvifrons*, **C.** *P*. *marginatus*, **D.** *P*. *babaulti* (including its junior synonym *P*. *pleurospilus*), **E.** *P*. *margaretae* (including localities mentioned in text) and **F.** symbols and colours used to denote collection sites of cichlids and of *Cichlidogyrus* specimens.

This study is an example of how *Cichlidogyrus* species may serve as complementary markers to investigate the taxonomy of their hosts (Fig 15). It also shows the potential of parasitology as an additional discipline in integrative taxonomy. Although many factors might obscur patterns in parasite assemblages [100–101], a certain degree of congruence between host and parasite interrelationships is often apparent [102]. The utility of *Cichlidogyrus* as additional markers in tropheine cichlids, including *Simochromis* and *Pseudosimochromis* (Figs 15 and 16), is no surprise as high host specificity has been shown in this system before [45]. Yet, *P*. *margaretae* was only collected once, and the *Cichlidogyrus* fauna infecting *Simochromis* and *Pseudosimochromis* consisted of species which were all new to science. This shows that even for well-studied models such as Lake Tanganyika cichlids, our basic understanding of biological diversity still depends on new discoveries.

## Appendix: Specimens Examined

Gill arches were drawn for specimens denoted with * (Fig 3)

### Simochromis diagramma

Morphology: BMNH 1889.1.30.9 (1), lectotype, 70.59 mm SL, Ujiji, Tanzania, coll. Coode-Hore; BMNH 1889.1.30.10–11 (2), paralectotypes, 52.08, 58.87mm SL, Ujiji, Tanzania, coll. Coode-Hore; MRAC 106503 (1), 79.84 mm SL, Kasanga, plage au sud de la rivière Kawa, Tanzania, 8° 28’ S 31° 9’ E, coll. M. Poll; MRAC 95-96-P-1377-90 (2), 66.32*, 67.15 mm SL, Ninde, Tanzania, 7° 40’ S 30° 43’ E, coll. exp. 95; MRAC B0-12-P-354-355 (2), 64.23, 95.01 mm SL, Cap Banza, Ubwari Peninsula, D.R.Congo, 4° 04’ S 29° 14’ E, coll. exp. 2010; MRAC B0-12-P-356-357 (1), Mugayo, D.R.Congo, 6° 47’ S 29° 33’ E, coll. exp. 2010; DNA barcoding: MRAC T95-24 (1), MRAC 95-96-P-2949 (1), Wampembwe, Tanzania, 8° 0’ S 30° 53’ E, coll. exp. 95; T95-1767 (1), Chaitika Point, Zambia, 8° 34’ S 30° 48’ E, coll. exp. 95; MRAC 92-81-P-1354 (1), Masaka Point, Tanzania, 5° 2’ S 29° 46’ E, coll. exp. 92; MRAC B0-12-P-354 (1), Cap Banza, Ubwari, D.R.Congo, 4° 4’ S 29° 14’ E, coll. exp. 2010; not included in analysis: NMW 24777–1 (1), lectotype of *Tilapia adolfi* Steindachner, 1909, 79.3 mm SL, Lake Tanganyika, coll. unknown; NMW 24777–2, 24778–81 (5), paralectotypes of *Tilapia adolfi* Steindachner, 1909, 56.4–67.6 mm SL, Lake Tanganyika, coll. unknown.

### Pseudosimochromis curvifrons

Morphology: MRAC 53101, holotype, 89.72 mm SL, Nyanza, Burundi, coll. A. Lestrade; MRAC 53102–53103 (2), paratypes, 84.47, 94.55 mm SL, Nyanza, Burundi, coll. A. Lestrade; MRAC B0-12-P-441-444 (3), 71.18, 74.54, 75.3 mm SL, Kyanza, D.R.Congo, 7° 7’ S 29° 59’ E, coll. exp. 2010; MRAC B0-12-P-445-448 (3), 66.59, 71.24, 79.70* mm SL, Kikoti, D.R.Congo, 7° 11’ S 30° 4’ E, coll. exp. 2010; DNA barcoding: MRAC 95-96-P-652 (1), Chaitika Point, Zambia, 8° 34’ S 30° 48’ E, coll. exp. 95; MRAC T95-1349 (1) Wampembwe, Tanzania, 8° 0’ S 30° 53’ E, coll. exp. 95.

### Pseudosimochromis babaulti

Morphology: MNHN 1927–318, holotype, 62.81 mm SL, Ouvira, D.R. Congo, 3° 24’ S 29° 8’ E, coll. G. Babault; MRAC 86560 (1), 60.4 mm SL, Kamvimvira, D.R.Congo, 3°21’ S 29° 9’ E, coll. Miss. Pisc. Katanga; MRAC 114401 (1), 114410 (1), 55.19, 57.73 mm SL, Uvira, D.R.Congo, 3° 24’ S 29° 8’ E, coll. G. Marlier; MRAC 74-19-P-8-9 (2), 64.07–78.72 mm SL, dans les environs de Bujumbura, 3° 23’ S 29° 22’ E, coll. RUCA; MRAC 92-81-P-200 (1), 79.08 mm SL, Ulwile Island, northern shore, Tanzania, 7° 28’ S 30° 34’ E, coll. exp. 92; MRAC 92-81-P-613 (1), 86.81 mm SL, Mpimbwe Hills, northern part of Shashete bay, Tanzania, 7° 7’ S 30° 30’ E, coll. exp. 92; MRAC 92-81-P-641-642 (2), 70.00–80.38 mm SL, just south of Kasinde, Tanzania, 7° 6’ S 30° 33’ E, coll. exp. 92; MRAC 92-81-P-716-719, 721–723, 727–728 (9), 53.81, 54.22, 56.81, 58.92, 59.10, 60.44, 66.54, 73.00, 76.15 mm SL, just south of Karema, Tanzania, 6° 51’ S 30° 27’ E, coll. exp. 92; MRAC 92-81-P-794-796 (3), 92-81-P-794-799 (1), 74.27, 77.12, 77.19, 80.88 mm SL, halfway between Ikola and Mkangasi, Tanzania, 6° 40’ S 30° 21’E, coll. exp. 92; MRAC 92-81-P-904 (1), 63.79 mm SL, Kalia, bay at mouth of Lugonesi River, Tanzania, 6° 27’ S 30° 8’ E, coll. exp. 92; MRAC 92-81-P-1150, 1053–1055, 1059 (5), 53.66, 62.86, 65.67, 68.08, 73.99 mm SL, Segunga, south of Segunga bay, Tanzania, 5°35’ S 29° 51’ E, coll. exp. 92; MRAC 92-81-P-1222 (1), 70.18 mm SL, Kalela, Tanzania, 5° 59’ S 29° 50’ E, coll. exp. 92; MRAC 92-81-P-1228 (1), 66.83 mm SL, 3 km north of Kabwe, Tanzania, 5°43’ S 29° 54’ E, coll. exp. 92; MRAC 95-96-P-643-645 (3), 95-96-P-3315 (1), 73.30, 75.29, 75.92, 80.71 mm SL, Msamba bay, Tanzania, 7° 51’ S 30° 47’ E, coll. exp. 95; MRAC 95-96-P-3317 (1), 66.91 mm SL, Kapele, Zambia, 8° 35’ S 31° 10’ E, coll. exp. 95; MRAC B0-12-P-424-425 (2), B0-12-P-394-398 (4), 61.95, 65.69, 67.10, 67.31, 74.15, 74.99 mm SL, Kyanza, D.R.Congo, 7° 07’ S 29° 58’ E; MRAC B0-12-P-411-417 (5), 68.12, 70.61, 72.55, 72.68, 74.38* mm SL, Mukamba, D.R.Congo, 6° 57’ S 29° 43’ E, coll. exp. 2010; MRAC 77-35-P-117 (1), holotype of *P*. *pleurospilus*, 78.80 mm SL, Chaitika, Zambia, 8° 34’ S 30° 48’ E, coll. P. Brichard; MRAC 77-35-P-121 (1), allotype of *P*. *pleurospilus*, 62.60 mm SL, Chaitika, Zambia, 8° 34’ S 30° 48’ E, coll. P. Brichard; MRAC 77-35-P-118-120 (3), paratypes of *P*. *pleurospilus*, 67.11, 75.07, 82.81 mm SL, Chaitika, Zambia, 8° 34’ S 30° 48’ E, coll. P. Brichard; MRAC 76-4-P-464, 465, 480 (3) paratypes of *P*. *pleurospilus*, 57.51, 65.10, 67.22 mm SL, Kama bay, Zambia, coll. P. Brichard; MRAC 76-4-P-95-96 (2), paratypes of *P*. *pleurospilus*, 68.44, 73.48 mm SL, Cap Kabayeye, à l’est de Kasaba bay, Zambia, coll. P. Brichard; MRAC 76-4-98-99 (2), paratypes of *P*. *pleurospilus*, 59.98, 61.25 mm SL, Cap Nundo, Zambia, 8° 31’S 30° 38’ E, coll. P. Brichard, MRAC 78-25-P-703-704 (1), 73.04 mm SL, Chaitika, Zambia, 8° 34’ S 30° 48’ E, coll. P. Brichard; DNA barcoding: MRAC 92-81-P-1355, 56 (2), Masaka Point, Tanzania, 5° 2’ S 29° 46’ E, coll. exp. 92; MRAC 92-81-P-1228 (1), 3 km north of Kabwe, Tanzania, 5°43’ S 29° 54’ E, coll. exp. 92; MRAC 92-81-P-726, 27 (2), just south of Karema, Tanzania, 6° 51’ S 30° 27’ E, coll. exp. 92; MRAC B0-12-P-812, 73 (2), Mufazi, D.R.Congo, 7° 05’ S 29° 55’ E, coll. exp. 2010; MRAC B0-12-P-420 (1), Mukamba, D.R.Congo, 6° 57’ S 29° 43’ E, coll. exp. 2010; MRAC B0-12-P-1094, 95 (2), Mtoto, D.R.Congo, 6° 58’ S 29° 44’ E, coll. exp. 2010; MRAC B0-12-P-841, 42 (2), Kapakwe, D.R.Congo, 6° 58’ S 29° 44’ E, coll. exp. 2010; MRAC B0-12-P-424, 25 (2), Kyanza, D.R.Congo, 7° 7’ S 29° 59’, coll. exp. 2010; MRAC B0-12-P-427, 28 (2), Kikoti, D.R.Congo, 7° 11’ S 30° 04’ E, coll. exp. 2010; as *P*. *pleurospilus*: MRAC T95-1314, 15 (2), Nakaku Village, Zambia, 8° 41’ S 30° 54’ E, coll. exp. 95; MRAC T95-1771 (1), Chaitika Point, Zambia, 8° 34’ S 30° 48’ E, coll. exp. 95.

### Pseudosimochromis margaretae

Morphology: SAIAB 966 (2), paratypes, 68.79*, 76.43 mm SL, Kigoma Harbour, Tanzania, coll. G. S. Axelrod, 4° 52’ S 29° 37’ E

### Pseudosimochromis marginatus

Morphology: MRAC 106523, holotype, 74.22 mm SL, Manga (Ubwari), plage et rive rocheuse, D.R.Congo, 4° 9’ S 29° 13’ E, coll. M. Poll; INRS 360 (2), paratypes, 51.53, 67.77 mm SL, Manga (Ubwari), plage et rive rocheuse, D.R.Congo, 4° 9’ S 29° 13’ E, coll. M. Poll; MRAC 106524–25 (2), paratypes, 67.03, 70.46 mm SL, Manga (Ubwari), plage et rive rocheuse, D.R.Congo, 4° 9’ S 29° 13’ E, coll. M. Poll; MRAC 129674–87 (5), 68.20, 71.68, 72.18, 77.67, 80.44 mm SL, Luhanga, D.R.Congo, 3° 31’ S 29° 9’ E, coll. G. Matthes; MRAC 82-12-P-157-158 (2), 67.78, 69.48 mm SL, Ubwari, face ouest, D.R.Congo, between 4° 07’ S 29° 15’E and 4° 31’ S 29° 13’ E, coll. M. Schreyen; MRAC 92-81-P-998, 1007, 1014* (3), 61.42, 74.48, 78.52 mm SL, 1/3 distance Bulu Point—Luagala Point, Tanzania, 6° 10’ S 29° 40’ E, coll. exp. 92; MRAC B0-12-P-375-379 (5), 67.44, 69.20, 72.15, 73.84, 80.34 mm SL, Lubumba, D.R.Congo, 3° 59’ S 29° 7’ E, coll. exp. 2010; MRAC B0-12-P-360 (2), 68.05, 71.94 mm SL, Cap Banza, Ubwari, D.R.Congo, 4° 4’ S 29° 14’ E, coll. exp. 2010; MRAC B0-12-P-361 (1), 67.16 mm SL, Kisokwe, D.R.Congo, 4° 15’ S 29° 11’ E, coll. exp. 2010; MRAC B0-12-P-382 (1), 80.49 mm SL, Magogoro, Kavala Islands, D.R.Congo, 5° 39’ S 29° 12’ E, coll. exp. 2010; MRAC B0-12-P-384-385 (2), 48.91, 52.48 mm SL, Mukindu, D.R.Congo, 5° 36’ S 29° 23’ E, coll. exp. 2010; MRAC B0-12-P-386-393 (8), 66.33, 66.07, 78.89, 77.60, 70.30, 69.78, 66.73, 67.65, Musinwa, D.R.Congo, 5° 41’ S 29° 25’ E, coll. exp. 2010; DNA barcoding: MRAC B0-12-P-361 (1), Kisokwe, D.R.Congo, 4° 15’ S 29° 11’ E, coll. exp. 2010; MRAC B0-12-P-384 (1), Mukindu, D.R.Congo, 5° 36’ S 29° 23’ E, coll. exp. 2010; MRAC B0-12-P-390 (1), Musinwa, D.R.Congo, 5° 41’ S 29° 25’ E, coll. exp. 2010; MRAC 92-81-P-998 (1), 1/3 distance Bulu Point—Luagala Point, Tanzania, 6° 10’ S 29° 40’ E, coll. exp. 92.

### Additional specimens used for DNA-barcoding

*Limnotilapia dardennii*: MRAC 92-81-P-285, Ulwile Island, northern shore, Tanzania, 7° 28’ S 30° 34’ E, coll. exp. 92; MRAC 95-96-P-2867, Wampembwe, Tanzania, 8° 0’ S 30° 53’ E, coll. exp. 95; 95-96-P-2866, Punda Point, Tanzania, 7° 27’ S 30° 36’ E, coll. exp. 95; ‘*Ctenochromis*’ *horei*: MRAC 92-81-P-618, Mpimbwe Hills, northern part of Shashete bay, Tanzania, 7° 7’ S 30° 30’ E, coll. exp. 92; MRAC 92-81-P-680, north of Mkombe, Tanzania, 6° 58’ E 30° 34’ S, coll. exp. 92; MRAC 92-81-P-1357, Masaka Point, Tanzania, 5° 2’ S 29° 46’ E, coll. exp. 92; ‘*Gnathochromis*’ *pfefferi*: MRAC 95-96-P-869, Chaitika Point, Zambia, 8° 34’ S 30° 48’ E, coll. exp. 95; MRAC 95-96-P-2817, Wampembwe, Tanzania, 8° 0’ S 30° 53’ E, coll. exp. 95; MRAC 92-81-P-1315, Kibwe Bay, Tanzania, 5° 24’ E 29° 46’ S, coll. exp. 92.

## Supporting Information

S1 File Morphometric data. Measurements and meristics taken on 114 specimens belonging to *Simochromis* and *Pseudosimochromis* and measurements taken on the specimens of *Cichlidogyrus raeymaekersi* **sp. nov.**, *C*. *muterezii* **sp. nov.**, *C*. *banyankimbonai* **sp. nov.**, *Cichlidogyrus georgesmertensi* **sp. nov.**, *C*. *franswittei* **sp. nov.** and *C*. *frankwillemsi* **sp. nov.**
(XLSX)
