# Correction: Morphology, Molecules, and Monogenean Parasites: An Example of an Integrative Approach to Cichlid Biodiversity

**DOI:** 10.1371/journal.pone.0129987

**Published:** 2015-06-03

**Authors:** The PLOS ONE Staff

The affiliation for the sixth author is not indicated. Maarten P. M. Vanhove is affiliated with: 1 Biology Department, Royal Museum for Central Africa, Tervuren, Belgium, 2 Laboratory of Biodiversity and Evolutionary Genomics, Department of Biology, University of Leuven, Leuven, Belgium, 5 Institute of Marine Biological Resources and Inland Waters, Hellenic Centre for Marine Research, Anavyssos, Greece, 6 Laboratory of Parasitology, Department of Botany and Zoology, Faculty of Science, Masaryk University, Brno, Czech Republic, ¤b Current address: Capacities for Biodiversity and Sustainable Development, Operational Directorate Natural Environment, Royal Belgian Institute of Natural Sciences, Brussels, Belgium. The publisher apologizes for the error.
